# Location of Parasympathetic Innervation Regions From Electrograms to Guide Atrial Fibrillation Ablation Therapy: An *in silico* Modeling Study

**DOI:** 10.3389/fphys.2021.674197

**Published:** 2021-08-11

**Authors:** Chiara Celotto, Carlos Sánchez, Konstantinos A. Mountris, Pablo Laguna, Esther Pueyo

**Affiliations:** ^1^Aragon Institute of Engineering Research-I3A-, University of Zaragoza, IIS Aragón, Zaragoza, Spain; ^2^CIBER in Bioengineering, Biomaterials and Nanomedicine, Zaragoza, Spain

**Keywords:** atrial fibrillation, autonomic nervous system, electrograms, computational simulation, achetylocholine, catheter ablation, ganglionated plexi, repolarization EGM

## Abstract

The autonomic nervous system (ANS) plays an essential role in the generation and maintenance of cardiac arrhythmias. The cardiac ANS can be divided into its extrinsic and intrinsic components, with the latter being organized in an epicardial neural network of interconnecting axons and clusters of autonomic ganglia called ganglionated plexi (GPs). GP ablation has been associated with a decreased risk of atrial fibrillation (AF) recurrence, but the accurate location of GPs is required for ablation to be effective. Although GP stimulation triggers both sympathetic and parasympathetic ANS branches, a predominance of parasympathetic activity has been shown. This study aims was to develop a method to locate atrial parasympathetic innervation sites based on measurements from a grid of electrograms (EGMs). Electrophysiological models representative of non-AF, paroxysmal AF (PxAF), and persistent AF (PsAF) tissues were developed. Parasympathetic effects were modeled by increasing the concentration of the neurotransmitter acetylcholine (ACh) in randomly distributed circles across the tissue. Different circle sizes of ACh and fibrosis geometries were considered, accounting for both uniform diffuse and non-uniform diffuse fibrosis. Computational simulations were performed, from which unipolar EGMs were computed in a 16 × 1 6 electrode mesh. Different distances of the electrodes to the tissue (0.5, 1, and 2 mm) and noise levels with signal-to-noise ratio (SNR) values of 0, 5, 10, 15, and 20 dB were tested. The amplitude of the atrial EGM repolarization wave was found to be representative of the presence or absence of ACh release sites, with larger positive amplitudes indicating that the electrode was placed over an ACh region. Statistical analysis was performed to identify the optimal thresholds for the identification of ACh sites. In all non-AF, PxAF, and PsAF tissues, the repolarization amplitude rendered successful identification. The algorithm performed better in the absence of fibrosis or when fibrosis was uniformly diffuse, with a mean accuracy of 0.94 in contrast with a mean accuracy of 0.89 for non-uniform diffuse fibrotic cases. The algorithm was robust against noise and worked for the tested ranges of electrode-to-tissue distance. In conclusion, the results from this study support the feasibility to locate atrial parasympathetic innervation sites from the amplitude of repolarization wave.

## 1. Introduction

The autonomic nervous system (ANS) controls all aspects of cardiac activity, including regulation of heart rate, atrio ventricular conduction, refractoriness, and contractility (Gatti et al., 1995; Dickerson et al., 1998; Gray et al., 2004; Hou et al., 2007). A multiple-level hierarchy has been described for the cardiac autonomic nervous system (CANS), including central components, intrathoracic extracardiac components and, intrinsic cardiac components. The extrinsic components of the CANS comprise brain or spinal conglomerations of neuron bodies, connected to the heart through their axons, while the intrinsic cardiac nervous system consists of a neural network formed by nerve axons, interconnecting neurons and clusters of autonomic ganglia called ganglionated plexi (GP) (Pauza et al., 2000; Stavrakis and Po, 2017). The shape and structure of the neural network in the myocardium are not fully known, but some studies have documented that between 500 and 1,500 ganglia of different sizes are present in the atrial and ventricular myocardium (Pauza et al., 2000). Ganglia may contain 200–1,000 neurons (Armour et al., 1997; Pauza et al., 2000; Choi et al., 2017), including sympathetic and parasympathetic efferent neurons, afferent neurons and local interneurons, that receive inputs from both efferent and afferent neurons. Studies have shown that GPs are important mediators in the interaction of the intrinsic CANS with the extrinsic CANS (Hadaya and Ardell, 2020).

Specifically, regarding the atria, four major atrial GPs have been reported to be embedded in epicardial fat pads located near the pulmonary veins (PVs), with each of them innervating one of the four PVs as well as the nearby atrial myocardium (Armour et al., 1997; Pauza et al., 2000). Although the vast majority of ganglion cells are cholinergic, most ganglia also contain adrenergic nerve fibers (Tan et al., 2006). Cholinergic neurons, contain choline acetyltransferase (ChAT), required for the synthesis of acetylcholine (ACh). ACh released by postganglionic cholinergic axons has an effect on the transmembrane potential of cardiomyocytes by activating the ACh-sensitive inward rectifier potassium current, I_*KACh*_, through G-protein coupled receptors (Rysevaite et al., 2011).

The ANS has been shown to play an important role in the pathophysiology of cardiac arrhythmias, including atrial fibrillation (AF) (Tai, 2001; Chen et al., 2006, 2014; Chen and Tan, 2007). The impact of autonomic tone variations in the development of paroxysmal AF (PxAF) has been described in human and animal studies (Bettoni and Zimmermann, 2002; Oliveira et al., 2011). Direct recordings of extrinsic and intrinsic CANS have allowed demonstrating the individual relevance of atrial GPs in AF. In a canine AF model of atrial tachycardia remodeling, autonomic ganglia are shown to be important for maintenance of AF. In ambulatory dogs, intrinsic CANS activity always precedes episodes of PxAF and paroxysmal atrial tachycardia (Choi Eue-Keun et al., 2010). Activation of the I_*KACh*_ current upon ACh release from cholinergic neurons in atrial GPs can lead to shortening of the action potential duration (APD) and the effective refractory period of atrial myocytes (Verkerk et al., 2012), which could be proarrhythmic. Primarily, since ACh is rapidly broken down at its release site by acetylcholinesterase, its effects can be largely spatially heterogeneous (Skibsbye et al., 2014), thus additionally contributing to increased AF vulnerability (Nattel et al., 2000; Chen et al., 2006, 2014); however, due to the presence of both adrenergic and cholinergic nerve structures in the intrinsic CANS, both the sympathetic and parasympathetic branches of the ANS could be contributing to the observed atrial arrhythmias.

Radiofrequency catheter ablation is one of the most common procedures for AF treatment when anti-arrhythmic drug therapy is not effective. Targets for successful AF ablation are continuously being sought (Hwang et al., 2016). GP ablation has been associated with a decreased risk of recurrence of AF (Pappone et al., 2004; Scherlag et al., 2005; Scanavacca et al., 2006; Pokushalov et al., 2009; Han et al., 2010; Katritsis et al., 2011; Mikhaylov et al., 2011; Mao Jun et al., 2014), either in addition to the pulmonary vein (PV) isolation (Scanavacca et al., 2006; Lin et al., 2007, 2008; Lemola et al., 2008; Po et al., 2009; Zhou et al., 2011; Katritsis et al., 2013) or as a stand-alone procedure (Po et al., 2009; Pokushalov et al., 2010). The percentage of success in eliminating AF seems to be similar for PV isolation and GP ablation individually, but it is significantly higher when the two are combined (Po et al., 2009; Katritsis et al., 2013; Stavrakis and Po, 2017). One potential contributing factor to the effectiveness of GP ablation is accurate GP location. During ablation procedures, GPs are normally located by the induction of vagal reflexes through rapid, short stimulation at their expected anatomical sites until a positive response is found (AV block, hypotension, or more than 50% increase in R-R interval during AF) (Lemery, 2006; Po et al., 2009; Scherlag et al., 2009; Choi et al., 2017). As an alternative to this functional approach, the anatomical approach consists of the ablation of the four main GPs based on their presumed anatomical locations (Katritsis et al., 2013). An important limitation of these methods is that once the GPs have been located, there is still no information on the actual area occupied by each of them (Stavrakis et al., 2015).

This study aims to develop a method to locate regions of atrial parasympathetic innervation sites within atrial GPs based on measurements from a multielectrode grid of electrograms (EGMs). This approach is expected to provide information not only on the approximate location but also on the dimensions of those sites. We hypothesize is that the EGM atrial repolarization wave amplitude could be representative of the presence of ACh release sites (Vigmond et al., 2005), as I_*KACh*_ activation is expected to accelerate phase 3 of the AP, leading to higher EGM repolarization amplitudes. Even if the effectiveness of GP ablation has shown to be higher in PxAF (Pokushalov et al., 2009; Chao et al., 2012), in this study, both PxAF and persistent AF (PsAF) were simulated, with PsAF models including electrical and structural AF-related remodeling.

## 2. Materials and Methods

### 2.1. Human Atrial Tissue Models

Two-dimensional (2D) human atrial tissue models of 5 × 5 cm, with and without fibrosis, were built. A square grid of 251 by 251 nodes was used, where each node in the grid took the properties of a cardiomyocyte or a fibroblast. The Courtemanche model (Courtemanche et al., 1998) was used to describe atrial myocyte electrophysiology while fibroblasts were described with the MacCannell model (Andrew MacCannell et al., 2007), an active model which includes four membrane ionic currents. Parasympathetic effects were incorporated into the Courtemanche model by including, I_*KACh*_ as defined in Kneller et al. (2002) with the updates proposed in Bayer et al. (2019). An additional simulation considering cell-to-cell variability was performed. In this study, we only considered variability in the conductances of the currents having the largest effects on the last stage of repolarization (i.e., APD at 90% repolarization), which corresponds to the repolarization wave we are interested in analyzing. According to the study by Sánchez et al. (2014), I_*K*1_ and I_*CaL*_ were the two currents having the largest influence on APD_90_ in the Courtemanche model, while other currents like I_*NaK*_, I_*to*_, I_*Kur*_ and I_*NaCa*_ had notably lower effects on APD_90_. For the conductances of I_*K*1_ and I_*CaL*_, a variation range between –30 and +30% in 15% steps was considered, thus leading to 25 ionic combinations, which were used to simulate cells with distinct characteristics.

**Figure 1:**
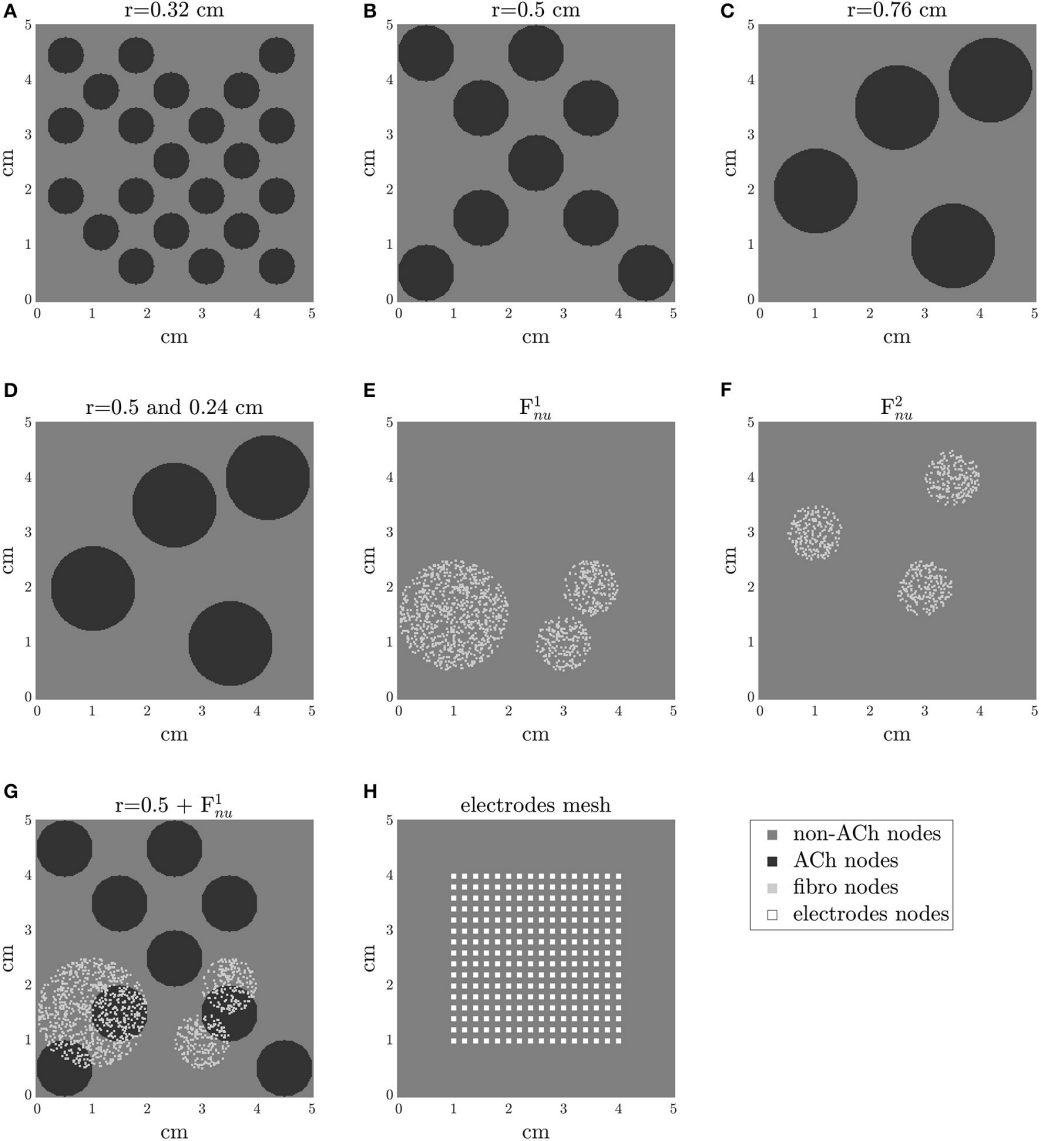
2D tissue models with different distributions of ACh release sites in **(A–D)** and with different fibrosis distributions in **(E,F)**. In **(G)**, an example of a tissue model with ACh release sites and non-uniform diffuse fibrosis (Fnu). In **(H)**, the mesh of electrodes is represented. Electrode size is adapted for clarity.

Non-AF, PxAF, and PsAF tissue models were developed. AF is usually a progressive disease, starting from short and infrequent episodes to longer and more frequent ones. In general, the progression from PxAF to PsAF forms is accompanied by alteration of the myocardial substrate. In these models, PsAF was characterized by both structural and electrical remodeling, whereas PxAF included only modest structural remodeling. One aspect of structural remodeling included in PxAF and PsAF tissue models was an increase in the amount of fibrosis with respect to the non-AF case, which was characterized by the absence of fibrosis (Boldt et al., 2004; Platonov et al., 2011). Fibrosis distribution in PxAF and PsAF tissues was modeled as either uniform diffuse (F_*u*_) or non-uniform diffuse (F_*nu*_) (de Jong et al., 2011). To generate a F_*u*_ distribution, each node in the tissue was assigned a probability of 0.2 (for 20% fibrosis) or 0.4 (for 40% fibrosis) of being a fibroblast, otherwise being an atrial cardiomyocyte. A F_*nu*_ distribution was defined by setting circular patches in the tissue and generating diffuse fibrosis within them in degrees of 20 or 40%. Two different geometries of the fibrotic patches were considered, denoted as ${\text{F}}_{nu}^{1}$ and ${\text{F}}_{nu}^{2}$, which are illustrated in Figures 1E,F.

Another aspect of structural remodeling in the PsAF tissue models was a reduction in the longitudinal diffusion coefficient (D) between myocytes to simulate the effects of gap junction remodeling (Kostin et al., 2002; Burstein Brett et al., 2009). D was varied to obtain longitudinal conduction velocities in line with values reported in the literature (Bayer et al., 2019). Values of D of 0.003 and 0.002 cm^2^/ms were considered for non-AF/PxAF and PsAF tissues, respectively. In all types of tissues, a transverse-to-longitudinal conductivity ratio of 0.5 was adopted. Fiber orientation was considered parallel to the *y*-axis. Furthermore, a 4-fold reduction in the diffusion coefficient was considered for myocyte-fibroblast and fibroblast-fibroblast coupling, both in PxAF and PsAF tissues (Krueger et al., 2013). Electrical remodeling was associated with PsAF only and was accounted for by reducing the maximal conductances of I_*to*_, I_*CaL*_, and I_*kur*_ by 50, 70, and 50%, respectively, as in Courtemanche (1999). Refer to Table 1 for a summary of simulation parameters.

**Table 1:** Characteristics of electrical and structural remodeling in non-atrial fibrillation (AF), paroxysmal-AF, and persistent AF (PsAF) tissues, and simulated ACh concentrations.

		**Non-AF**	**PxAF**	**PsAF**
Structural remodeling	Fibrosis (F_*u*_ or F_*nu*_)	0%	20%	40%
	D (cm^2^/ms) at myocyte-myocyte	0.003	0.003	0.002
	D reduction factor at myocyte-fibroblast		∝0.25	∝0.25
	Transverse-to-longitudinal conductivity ratio	0.5	0.5	0.5
Electrical remodeling	I_*to*_	∝1	∝1	∝0.5
	I_*CaL*_	∝1	∝1	∝0.3
	I_*kur*_	∝1	∝1	∝0.5
Parasympathetic effects		**Non-ACh sites**	**ACh sites**
	ACh (μM)	0	0.1

Parasympathetic effects were modeled by randomly increasing the concentration of the ACh neurotransmitter in circles distributed across the tissue, which activated the I_*KACh*_ current in those areas. We simulated an ACh dose of 0.1 μM, following a previous study in which ACh doses varying from 0 to 0.1 μM were used (Bayer et al., 2019). Cases of parasympathetically innervated circles all of the same radius, this being 0.32, 0.5, or 0.76 cm, as well as a case of parasympathetically innervated circles of different radii (0.24 and 0.5 cm) were considered. All these ACh geometries are illustrated in Figures 1A–D.

The interaction between vagal stimulation and fibrosis was investigated by considering models with all different combinations of ACh patches, 20–40% of F_*u*_ or F_*nu*_. One of these possible combinations is represented in Figure 1G. In the GPs, despite the predominance of the parasympathetic fibers, the sympathetic fibers can be found too (Tan et al., 2006). To account for it, an additional simulation considering small islands of β-adrenergic stimulation inside the ACh patches was performed (radius of Isoproterenol (Iso) patches = 0.1 cm). β-adrenergic stimulation was simulated as in the study by González de la Fuente et al. (2013), where the effects of Iso were modeled by the modulation of the maximum conductances of the I_*CaL*_, I_*to*_ and I_*Ks*_ currents following the reported dose-dependent curves. A 1μM Iso dose was considered.

### 2.2. Simulation Protocols

Tissue simulations were performed using Electra, an in-house software implementing the finite element and meshfree mixed collocation methods (Mountris et al., 2019; Mountris and Pueyo, 2020b) for the solution of the cardiac monodomain model. In this study, the finite element implementation was used as in previous studies, where we have simulated human atrial electrophysiology (Celotto et al., 2020). Simulations were performed using explicit time integration with an adaptive time step ranging from 0.005 to 0.01 ms. A dual adaptive explicit time integration (DAETI) method was used (Mountris and Pueyo, 2020a). DAETI employs adaptive explicit integration for the solution of both the reaction and diffusion terms of the cardiac monodomain model, which allows obtaining accurate solutions while reducing the computational time. In all tissue simulations, a space step of 0.02 cm was used. To define the spatial resolution, a convergence analysis was conducted by running simulations with progressively reduced space step. When differences in longitudinal conduction velocity between consecutive step sizes were below 0.2%, no improvement was considered to be achieved by additionally refining the mesh and that space step was taken as the spatial resolution for all the simulations.

To ensure that steady-state was reached in our simulations, single cells were simulated in MATLAB while being paced at a fixed cycle length (CL) of 1,000 ms for 1 min. The values of the state variables of the models at steady-state were used for initialization in the tissue simulations. Tissue stimuli were applied at its bottom edge, with a CL of 1,000 ms. The results for the last simulated beat were analyzed.

Simulations were run in a laptop with 8 GB of RAM and an Intel Core i7 4-core processor at 2.5 GHz.

### 2.3. Unipolar EGM Computation

Unipolar EGMs were computed in a 16 × 16 electrode mesh at the center of the tissue, with an inter-electrode spacing of 2 mm in both the “*x*” and “*y*” directions, as represented in Figure 1H. Electrode positioning at different distances from the tissue in the orthogonal direction, namely 0.5, 1, and 2 mm, was tested. To compute the EGMs $\phi_{e}\left({\text{r}}^{\prime },t\right)$ for an electrode located at **r**′, the following formula was used (Gima and Rudy, 2002; Baher et al., 2007; Palacio et al., 2019):

(1) $$\begin{array}{r} \phi_{e}\left({\text{r}}^{\prime },t\right)=\int \int \left[-\nabla_{\text{r}}V_{m}\left(\text{r},t\right)\cdot \nabla_{\text{r}}\left(\frac{1}{d\left(\text{r},{\text{r}}^{\prime }\right)}\right)\right]dx\;dy \end{array}$$

(2) $$\begin{array}{r} d\left(\text{r},{\text{r}}^{\prime }\right)=||\text{r}-{\text{r}}^{\prime }|{|}_{2} \end{array}$$

where **r** = [*x* *y* *z*] and **r**′ = [*x*′ *y*′ *z*′] are the coordinate vectors, in the Cartesian system, for a tissue point and the electrode, respectively. In the equation, ∇_**r**_ denotes the spatial gradient. The integral was calculated across the whole 2D tissue in the x-y plane. The discretized form of this formula is reported in the Supplementary Material.

$\phi_{e}\left({\text{r}}^{\prime },t\right)$ represents the unipolar electrogram for a “punctual” electrode (pEGM) located at **r**′. In addition, to model the electrode size more realistically, electrodes of 0.8 × 0.8 mm (Chouvarda et al., 2001; Sahli Costabal et al., 2018; Abdi et al., 2020) were considered. For “dimensional” electrodes, the EGM correspondent to that electrode was obtained by performing the average of the pEGMs computed in all the points covered by the surface of the electrode, namely 25 nodes (Abdi et al., 2020). The EGMs were then normalized by a factor dependent on the electrode-tissue distance (350 for *z* = 0.5 mm, 250 for *z* = 1 mm and 150 for *z* = 2 mm) to obtain an amplitude of the depolarization wave in line with the amplitude of clinical EGMs (6/7 mV). Subsequently, the EGMs were filtered with a 2 Hz high-pass filter, mimicking the implementation in most commercial systems, to remove the DC level due to the differences in the resting membrane potentials between distinct cells in the tissue.

To assess the performance of the proposed algorithms (refer to section 2.4) under noisy conditions, noisy EGM signals were obtained as follows. Noise segments were extracted from 180 EGM recordings from patients. The power of each noise segment was normalized. Subsequently, the different noise segments were individually added to the EGMs with different multiplying factors to test signal-to-noise ratios (SNR) from 0 to 20 dB. For repolarization analysis, a 2 to 30 Hz band-pass filter was applied to the noisy EGMs to remove the DC level and the high frequency noise without altering the features of the repolarization waves. For depolarization analysis, a 2 to 250 Hz band-pass filter was applied.

### 2.4. Algorithm for Electrogram Post-processing

The atrial repolarization wave of the $\phi_{e}\left({\text{r}}^{\prime },t\right)$ signal, at the electrode (*i,j*), *i,j*∈{1, ⋯ , 16}, was denoted as *R*_*i,j*_(*t*). Since the time window (TW) in which the *R*_*i,j*_(*t*) waves were located did not show a fixed distance from the depolarization wave, an automatic delineation method was developed. For each simulated case, the time location, *t*_R_, of the absolute maximum repolarization peak (either positive or negative), *R*_*i*_*m*_, *j*_*m*__(*t*_R_), identified at the electrode (*i*_*m*_, *j*_*m*_) among all the (*i,j*) EGMs of a single case, was searched for in a window starting 100 ms after the depolarization-based reference time corresponding to the maximum slope of the depolarization wave, taken as *t* = 0. The onset and ending points of the TW were set to 30 ms before and 150 ms after the *t*_R_, respectively. For each EGM, the local minima ($R_{i,j}^{\text{min}}$) and maxima ($R_{i,j}^{\text{max}}$) within the TW were identified. The amplitude $R_{i,j}^{A}$ was selected as either $R_{i,j}^{\text{min}}$ or $R_{i,j}^{\text{max}}$, choosing the one with the largest absolute value (but maintaining its sign).

(3) $$\begin{array}{r} R_{i,j}^{A}=\arg\underset{x}{\max}\left\{\left|x\right|\right\},\;x\in \left\{R_{i,j}^{\text{min}},R_{i,j}^{\text{max}}\right\} \end{array}$$

**Figure 2:**
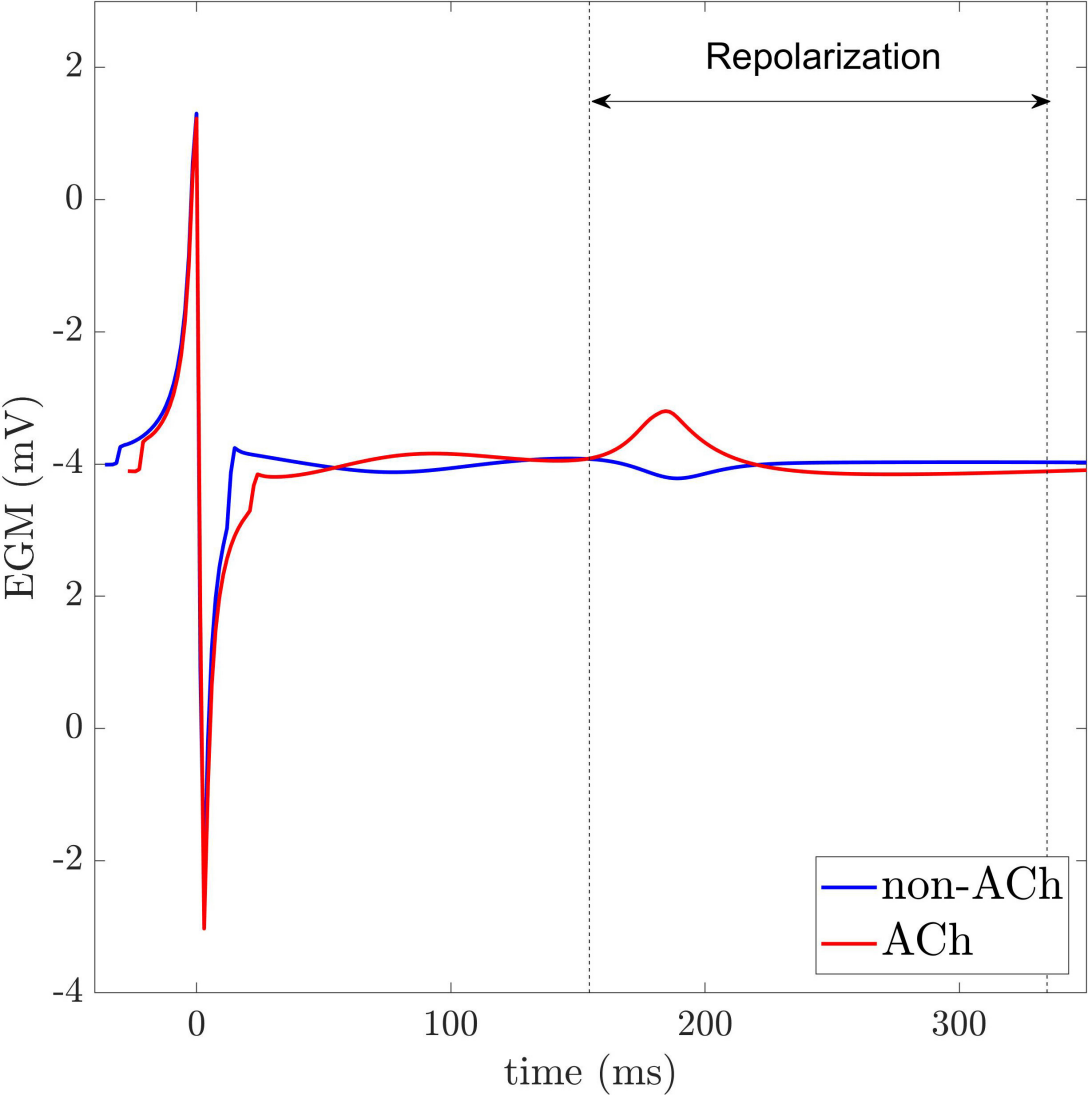
EGMs recorded in non-ACh (blue) and ACh (red) release sites. Dashed vertical lines indicate the repolarization window for analysis.

**Figure 3:**
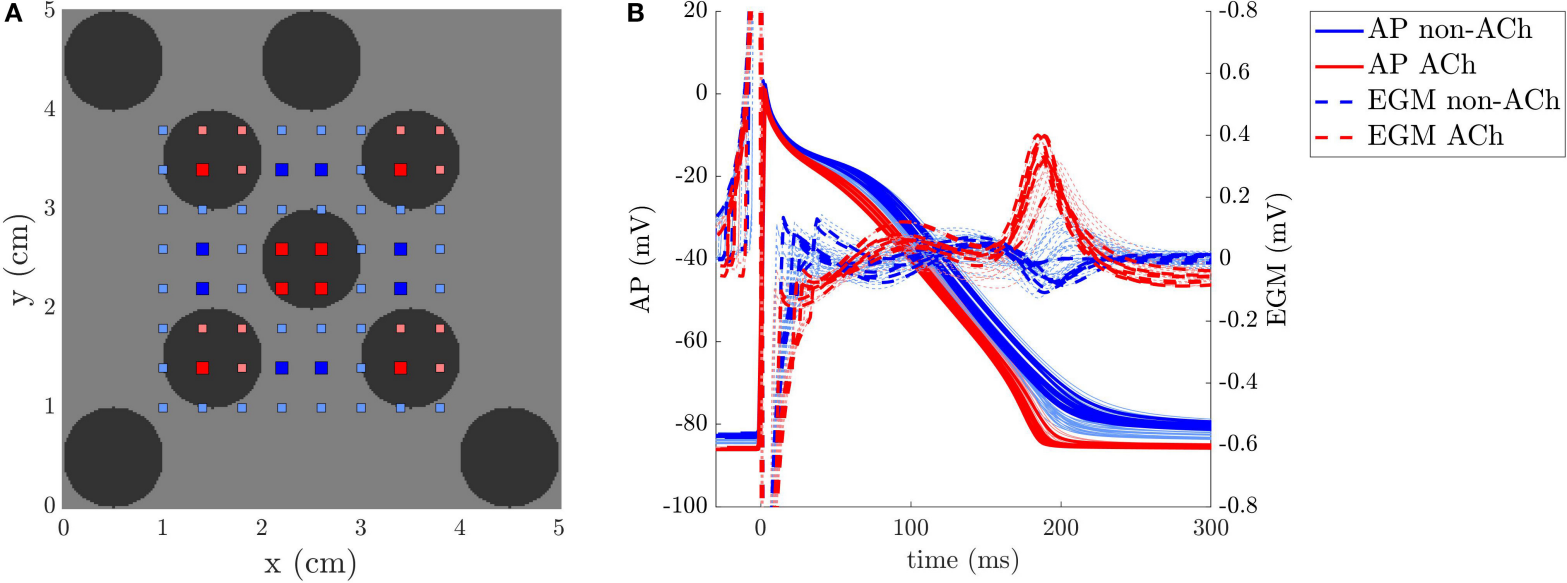
**(A)** 2D model of a non-atrial fibrillation (AF) tissue with ACh release sites shown in black and EGM electrodes in red and blue. **(B)** APs and EGMs were recorded in the (*i,j*) points represented in the left panel. The thicker lines correspond to the points represented with big squares in the tissue.

One example of two EGMs recorded inside and outside a GP, respectively, is represented in Figure 2. The relationship between the repolarization waves *R*_*i,j*_(*t*) and the corresponding APs are illustrated in Figure 3 for a simulated case with no fibrosis and with ACh distributed in patches of radius *r* = 0.5 cm. As it can be observed from the figure, ACh not only shortens the AP, but also accelerates phase 3 of AP repolarization, which leads to *R*_*i,j*_(*t*) waves of higher amplitude $R_{i,j}^{A}$. A sensitivity (Se)-specificity (Sp) analysis was performed by calculating a ROC curve to identify the optimal repolarization amplitude threshold, *R*^th^ that allowed identification of EGM signals of ACh-release and non-ACh-release areas. Specifically, ACh and non-ACh areas were associated with EGMs presenting amplitudes $R_{i,j}^{A}$ above and below the threshold *R*^th^, respectively.

**Figure 4:**
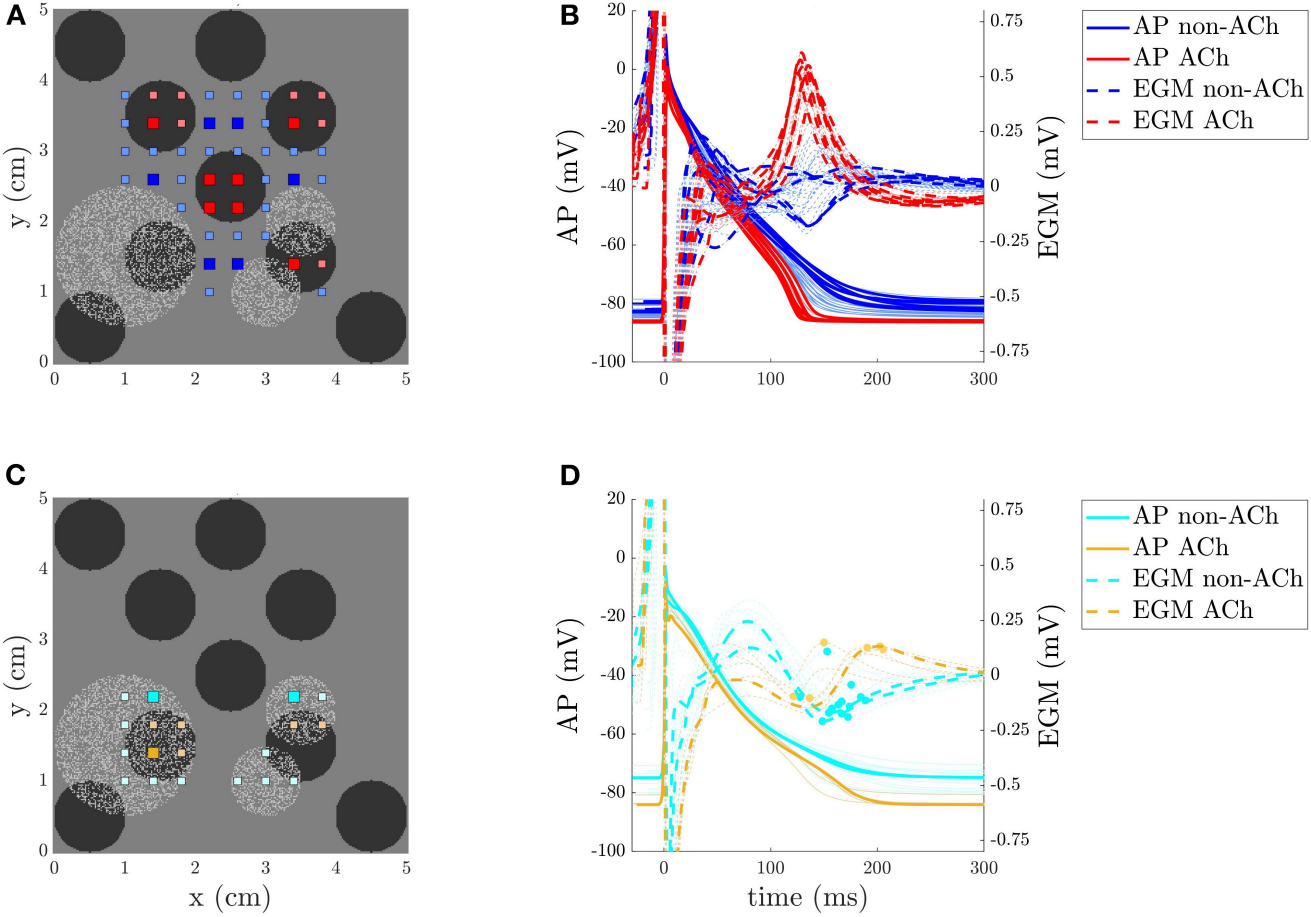
**(A,C)** 2D model of a persistent AF (PsAF) tissue with ACh release sites shown in black, fibrosis in light gray, and EGM electrodes in red and blue in the non-fibrotic regions, and in orange and cyan in the fibrotic regions. **(B,D)** APs and EGMs were recorded in the points represented in the left panels. The thicker lines correspond to the points represented with the big squares in the tissue. In **(D)** the peaks of the *R*_*i,j*_(*t*) waves are highlighted with a dot.

When simulating cases with F_*nu*_, we observed that the amplitude $R_{i,j}^{A}$ of EGMs in ACh and non-ACh areas presented different behavior depending on the presence or absence of fibrosis. This is illustrated in Figure 4 together with the different APs that underlie this difference. On this basis, an additional step in the EGM processing was applied to distinguish fibrotic from non-fibrotic areas before using the $R_{i,j}^{A}$ amplitude to identify ACh areas. Identification of fibrotic areas was performed based on the amplitude of the EGM depolarization wave, *D*_*i,j*_(*t*), taken from EGM onset to TW window onset, which is a common method in clinical practice (Rolf et al., 2014; Blandino et al., 2017; Nairn et al., 2020). The amplitude $D_{i,j}^{A}$ was computed as the difference between the maximum positive, $D_{i,j}^{\text{max}}$, and minimum negative, $D_{i,j}^{\text{min}}$, peaks of the depolarization wave *D*_*i,j*_(*t*).

(4) $$\begin{array}{r} D_{i,j}^{A}=D_{i,j}^{\text{max}}-D_{i,j}^{\text{min}} \end{array}$$

Fibrotic areas were associated with lower $D_{i,j}^{A}$ amplitudes. Considering the dependence of the depolarization amplitude on the amount of fibrosis, a ROC curve was again used to determine the optimal threshold *D*^th^ to distinguish fibrotic vs. non-fibrotic areas.

## 3. Results

### 3.1. EGM Repolarization Analysis in Non-AF, PxAF, and PsAF Tissues

In this section, the values of $R_{i,j}^{A}$ for ACh-release and non-ACh-release areas are presented. To illustrate the results, figures are presented for some of the simulated cases with ACh patches of radius *r* = 0.5 and ACh patches of radii *r* = 0.5 and 0.24 cm. Figures for all other simulated cases can be found in the Supplementary Figures 1–7.

In non-AF tissues, and for all the tested ACh geometries, the optimal value of the threshold *R*^th^ to distinguish between ACh and non-ACh regions, and computed as later described, was found to lie in a range between 23 and 41% of *R*^*A*, max^, the maximum $R_{i,j}^{A}$ value in the grid is given by the following:

(5) $$\begin{array}{r} R^{A,\text{max}}=\underset{i,j}{\max}\left\{R_{i,j}^{A}\right\}=R_{i_{m},j_{m}}\left(t_{\text{R}}\right). \end{array}$$

**Figure 5:**
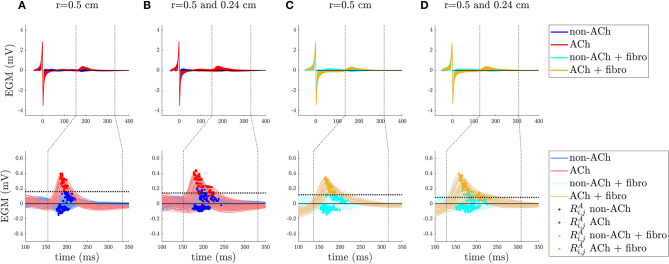
EGM analysis for a non-AF tissue in **(A,B)** and a peroxysmal-AF (PxAF) tissue with 20% uniform diffuse fibrosis (Fu) in **(C,D)**. Top row: EGMs aligned with respect to the time correspondent to the maximum slope of the depolarization wave (marked as *t* = 0 in the *x*-axis). The vertical dashed lines delimit the time window TW for the *R*_*i,j*_(*t*) repolarization signals. Bottom row: atrial repolarization waves, *R*_*i,j*_(*t*), with dots indicating the maximum absolute value, $R_{i,j}^{A}$, of the waves within TW. The horizontal dotted lines represent the optimal threshold *R*^th^ found by Se/Sp analysis.

In PxAF tissues with 20% F_*u*_ fibrosis, similar behavior as in non-AF tissues was found. Results are illustrated in Figure 5.

**Figure 6:**
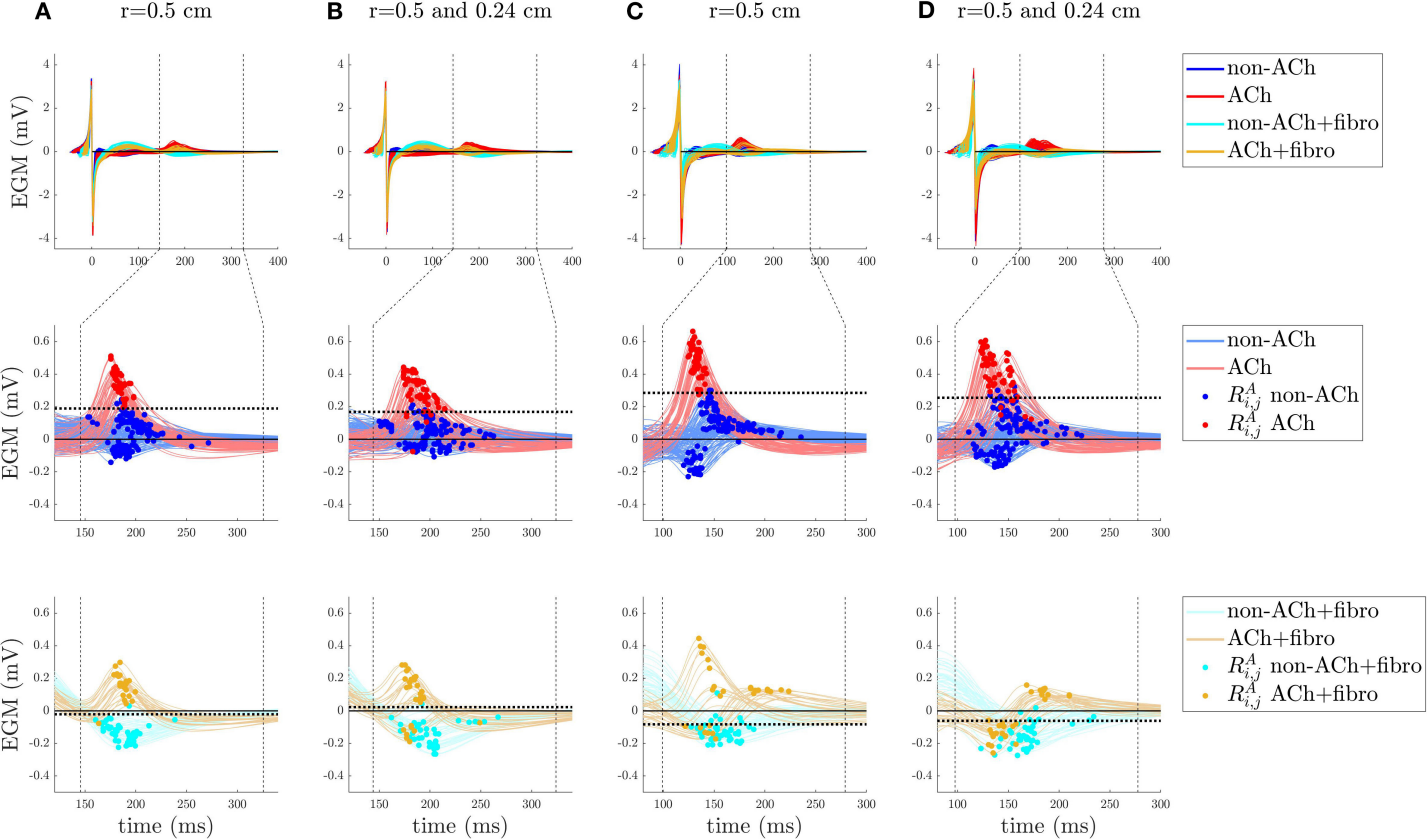
EGM analysis for a PxAF tissue with 20% non-uniform diffuse type 1 fibrosis in **(A,B)** and a PsAF tissue with 40% type 1 Fnu in **(C,D)** (type 1 Fnu is represented in Figure 1E) cases. Top row: EGMs aligned with respect to the time correspondent to the maximum slope of the depolarization wave (marked as *t* = 0 in the *x*-axis). The vertical dashed lines delimit the time window TW for the *R*_*i,j*_(*t*) repolarization signals. Bottom rows: atrial repolarization waves, *R*_*i,j*_(*t*), with dots indicating the maximum absolute value $R_{i,j}^{A}$ of the waves within TW. The horizontal dotted lines represent the optimal threshold *R*^th^ found by Se/Sp analysis.

In PxAF tissues with 20% F_*nu*_ fibrosis, the areas with fibrosis were analyzed separately from the areas without fibrosis. In the non-fibrotic regions, the threshold was found to lie in the range between 30 and 46% of *R*^*A*, max^ value. In the fibrotic regions, the peaks were generally organized in to two clusters above and below zero, with the positive peaks corresponding to ACh regions. The results of the analysis for 20% F_*nu*_ fibrosis with the simulated fibrotic geometry ${\text{F}}_{nu}^{1}$ are illustrated in Figure 6 for ACh patches of radius *r* = 0.5 cm and for ACh patches of radii *r* = 0.5 and 0.24 cm.

In PsAF tissues with 40% F_*u*_ fibrosis, similar to the previous cases, the threshold was found to lie in the range between 14 and 32% of *R*^*A*, max^. For PsAF tissues with 40% F_*nu*_ fibrosis, the behavior was the same as for 20% F_*nu*_ fibrosis reported above, with the only difference being the onset of the TW. Figure 6 shows the results for 40% F_*nu*_ fibrosis with the simulated fibrotic geometry ${\text{F}}_{nu}^{1}$.

### 3.2. EGM Depolarization Analysis in PxAF and PsAF Tissues

**Figure 7:**
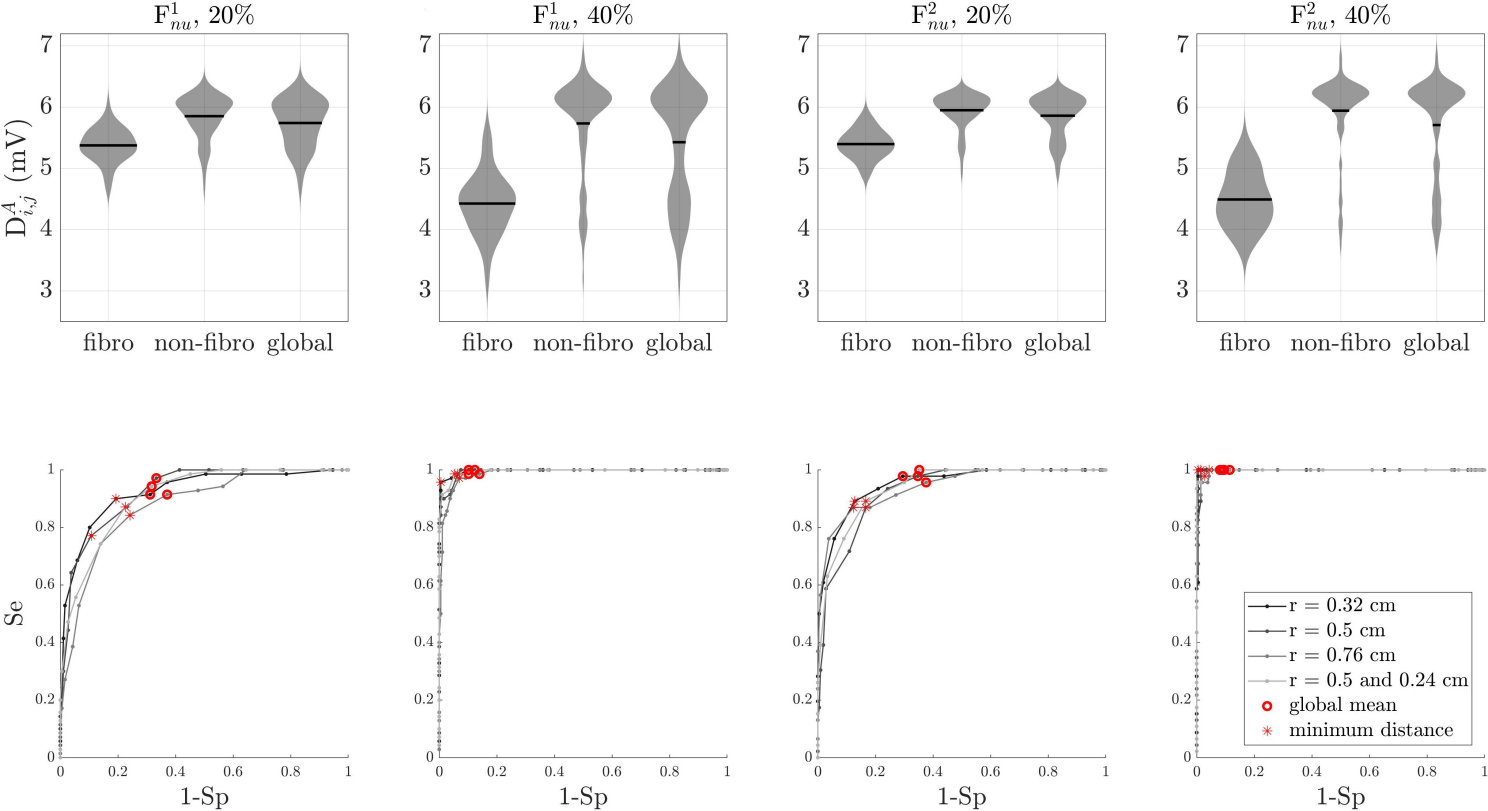
Depolarization wave, *D*_*i,j*_(*t*) analysis. Top row: statistical distribution of depolarization wave amplitude $D_{i,j}^{A}$ in fibrotic areas, non-fibrotic areas, and the whole tissue for simulated cases with non-uniform diffuse type 1 and type 2 fibrosis at 20 and 40%. Black lines represent the mean of the distribution. Bottom line: ROC curves for the same simulated cases as in the top row. Optimal thresholds minimizing the Euclidean distance to the top-left corner of the graph are shown with asterisks. Red circles correspond to the mean of the depolarization wave amplitudes. In all cases, the optimal threshold *D*^th^ is lower than the global mean.

Given the different morphology of the *R*_*i,j*_(*t*) waves in fibrotic vs. non-fibrotic areas when non-uniform diffuse fibrosis is simulated, prior identification of fibrotic areas was required to set up thresholds on repolarization amplitude that allow identification of ACh areas. The distribution of the depolarization amplitude $D_{i,j}^{A}$ in fibrotic areas, non-fibrotic areas, and the whole tissues are shown in Figure 7, top line. The results presented in Figure 7 for the ACh patches of radius 0.5 cm are representative of all other simulated cases, as ACh distribution does not have an observable effect on the amplitude of the depolarization waves. As it can be observed from the figure, although there is some overlap of the violin plots, particularly for 20% F_*nu*_ fibrosis, it is still possible to some extent to distinguish fibrotic vs. non-fibrotic areas based on the $D_{i,j}^{A}$ only.

### 3.3. Optimal Thresholds for Identification of Fibrotic and ACh-Release Areas

The optimal value for the threshold *D*^th^ on the depolarization amplitude to identify fibrotic areas was found by calculating a ROC curve. Starting from the global mean value of $D_{i,j}^{A}$, 40 different threshold values were analyzed by decreasing and increasing it in voltage steps of 0.1 mV. The optimal value for *D*^th^ was determined as the point on the curve that was closer, in terms of the Euclidean distance, to the top left corner corresponding to 100% sensitivity and 100% specificity. ROC curves for different fibrosis distributions are illustrated in Figure 7, bottom line. Overall, the optimal threshold value was lower than the global mean of $D_{i,j}^{A}$. From the ROC curves, it is also evident that the separability is higher for 40% F_*nu*_ than for 20% F_*nu*_ fibrosis distributions. The values of the threshold *D*^th^ on the depolarization amplitude are presented in Table 2, as % of the maximum $D_{i,j}^{A}$ value in the gridis given:

(6) $$\begin{array}{r} D^{A,\text{max}}=\underset{i,j}{\max}\left\{D_{i,j}^{A}\right\}. \end{array}$$

**Figure 8:**
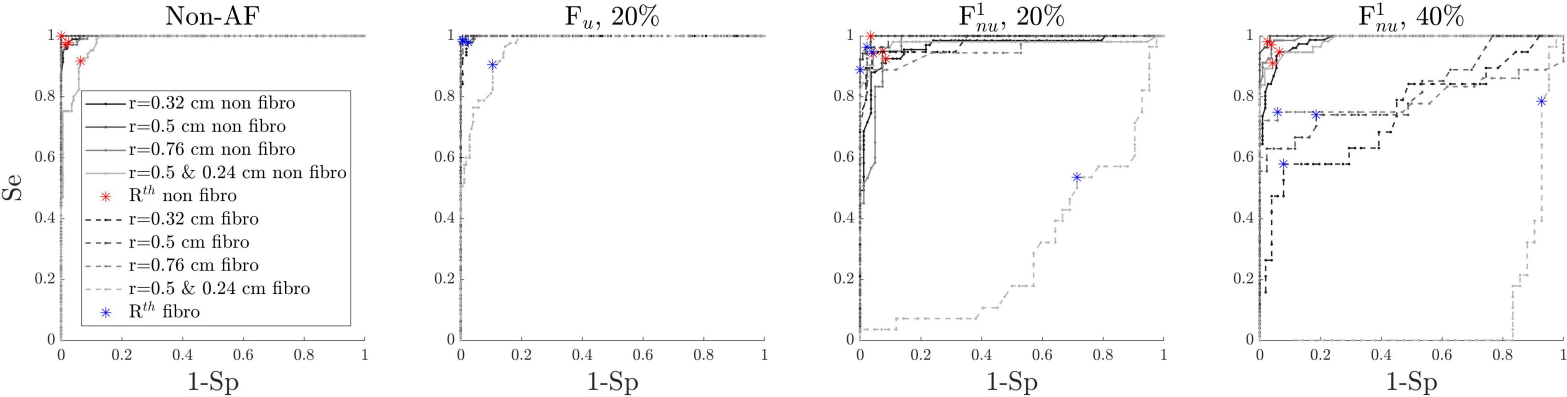
Repolarization wave analysis. ROC curves for non-AF tissues and PxAF and PsAF tissues with 20% uniform diffuse or type 1 fibrosis and 40% type 1 fibrosis. Results for different sizes of ACh sites are presented. Optimal thresholds *R*^th^ thresholds are shown in red and blue. For Fnu F_*p*_, cases, the curves for both the fibrotic (dashed lines) and non-fibrotic (continuous lines) regions are represented in the same plot.

The optimal value for the threshold *R*^th^ on the repolarization amplitude to identify ACh-release areas was analogously found by a statistical ROC curve analysis. Different thresholds of about 160 expressed in terms of percentage of *R*^*A*, max^, in voltage steps of 1% of *R*^*A*, max^, were studied. For simulated cases with F_*nu*_ distributions, the analysis was separately performed for fibrotic and non-fibrotic areas as previously identified according to the optimal threshold *D*^th^ described above. The ROC curves for ACh identification are illustrated in Figure 8. In the F_*nu*_ cases, ROC curves for both fibrotic and non-fibrotic areas are represented. As can be observed from the figure, the detection of ACh areas was more challenging in the fibrotic regions, especially when simulating 40% F_*nu*_ fibrosis. Furthermore, for both 20 and 40% F_*nu*_ fibrosis, the mixed case containing ACh patches of radii 0.5 and 0.24 cm has the worst measure of separability. The optimal values for the threshold *R*^th^ are displayed as dotted lines in Figures 5, 6. The values of *R*^th^ for all simulated cases are represented in Table 3.

**Table 2:** Values of the threshold *D*^th^ expressed as percentage of *D*^*A*, max^.

**r (cm)**	**${\text{F}}_{nu}^{1}$ 20%**	**${\text{F}}_{nu}^{2}$ 20%**	**${\text{F}}_{nu}^{1}$ 40%**	**${\text{F}}_{nu}^{2}$ 40%**	**Mean**
0.32	88	86	75	75	81
0.5	86	88	77	78	82
0.76	88	84	77	81	83
0.5; 0.24	87	87	80	78	83
Mean	87	86	77	78	82

**Table 3:** Value of the threshold *R*^th^ expressed as percentage of *R*^*A*, max^.

				**Non-fibrotic (fibrotic) regions**				
**r (cm)**	**Non-AF**	**_***u***_ 20%**	**_***u***_ 40%**	**${\text{F}}_{nu}^{1}$ 20%**	**${\text{F}}_{nu}^{2}$ 20%**	**${\text{F}}_{nu}^{1}$ 40%**	**${\text{F}}_{nu}^{2}$ 40%**	**Mean**
0.32	23	20	14	33 (–24)	33 (–67)	27 (–24)	30 (–28)	26 (–45)
0.5	41	34	32	36 (–16)	46 (–54)	43 (–23)	44 (+27)	39 (–35)
0.76	32	28	25	39 (–13)	33 (–50)	25 (–12)	44 (–11)	32 (–16)
0.5; 0.24	32	19	17	37 (+17)	30 (–59)	42 (–2)	42 (–16)	31 (–21)
Mean	32	25	22	36 (–9)	35 (–57)	34 (–19)	40 (–7)	32 (–33)

### 3.4. Identification of ACh-Release Areas From EGM Signals

**Figure 9:**
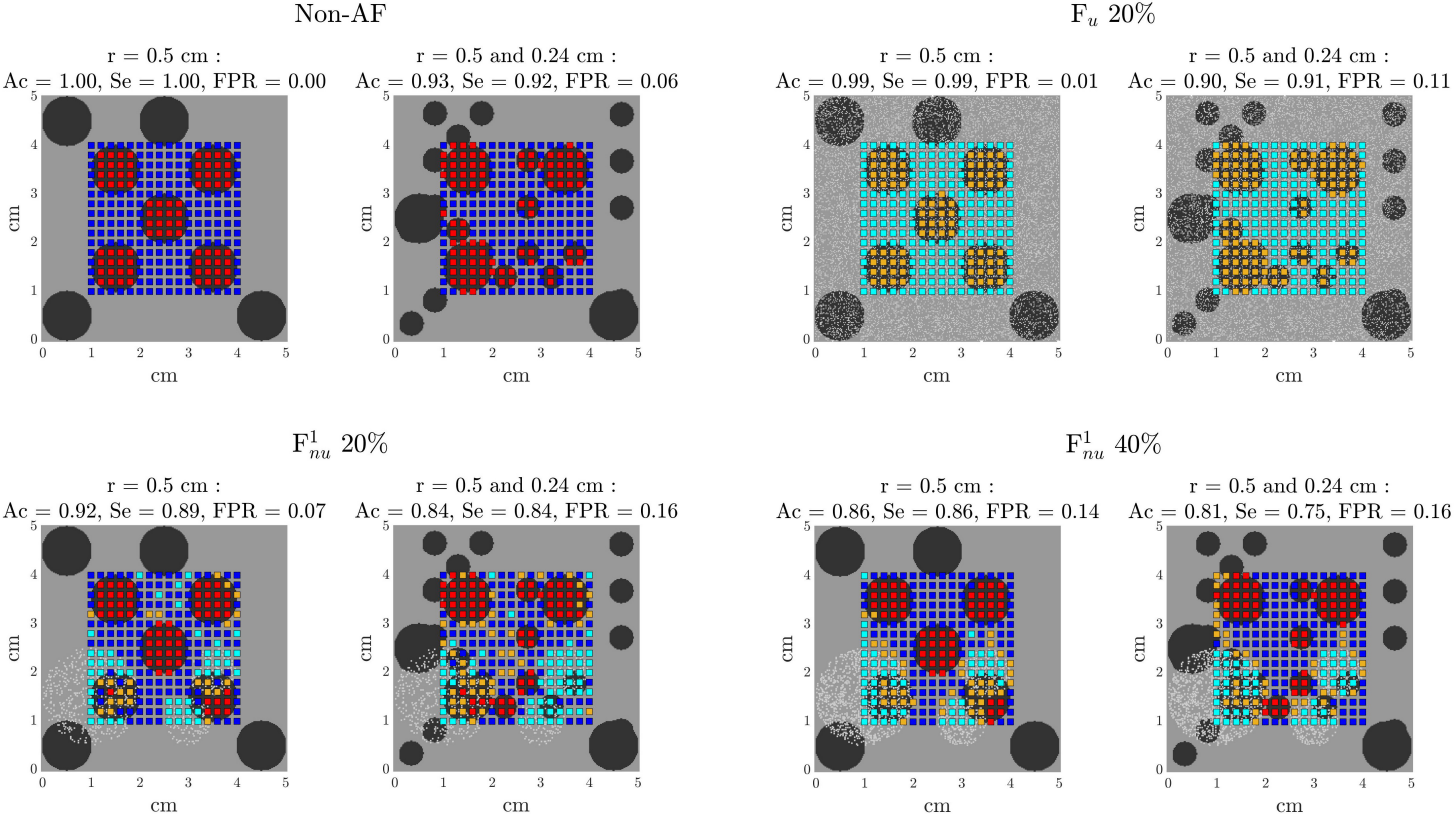
Results of the algorithm for detection of ACh release sites. Each electrode is assigned with non-ACh, ACh, non-ACh + fibro, or ACh + fibro on the basis of EGM analysis. The color code is the same as in Figures 5, 6.

The results on the identification of ACh-release areas are presented in Figure 9 for the simulated cases shown in Figures 5, 6 and other simulated cases can be found in the Supplementary Figures 8–10. The values of accuracy (Ac), sensitivity (Se), and false positive rate (FPR), equivalent to 1-Specificity, are reported above the maps presented in Figure 9. All results were obtained with the optimal values for the thresholds *D*^th^ and *R*^th^ described in section 3.3, except for the results presented in section 3.4.4 where the impact of the selected threshold is evaluated.

#### 3.4.1. Non-atrial Fibrillation Tissues

In non-fibrotic tissues, this algorithm was able to identify all the ACh-release patches, with similar performance measures for the different simulated cases. The the mean Ac and Se were both equal to 0.97 while mean FPR was equal to 0.03. The minimum Ac (0.93) and Se (0.92) and the maximum FPR (0.06) were obtained for ACh patches of different radii (*r* = 0.5 and *r* = 0.24).

#### 3.4.2. Paroxysmal-AF Tissues

In PxAF tissues with 20% F_*u*_, this algorithm was able to identify all the ACh patches, but, in the mixed case with ACh patches of different sizes, the isolated smaller areas were not completely identified. In PxAF tissues with ${\text{F}}_{nu}^{1}$ and ${\text{F}}_{nu}^{2}$, the algorithm showed very good performance (mean Acc = 0.83, mean FPR = 0.19), although some of the ACh patches in the fibrotic areas were not detected correctly in its whole extent. Furthermore, some of the sites wrongly detected as fibrotic were subsequently erroneously classified as ACh points.

However, in all cases, Ac and Se were above 0.80 and 0.83, respectively, and the maximum FPR rate was 0.22. F_*nu*_ cases presented worse performance than F_*u*_ cases, as can be observed in the representative cases presented in Figure 9.

#### 3.4.3. Persistent AF Tissues

In PsAF tissues with 40% F_*u*_, this algorithm was able to identify all the ACh patches. In PsAF tissues with ${\text{F}}_{nu}^{1}$ and ${\text{F}}_{nu}^{2}$, most of the ACh patches were successfully detected, but those that were of small size and fell completely inside a fibrotic area could not be detected. As in PxAF tissues, the mixed case with ACh patches of different sizes were the one presenting the worst performance.

In all cases, Ac and Se were above 0.84 and 0.82, respectively, The maximum FPR rate was 0.18. ACh identification in PsAF tissues with F_*nu*_ achieved superior performance than in PxAF tissues, mainly because the fibrotic regions were detected better. This can be appreciated from Figure 9, which shows the results for a PsAF tissue with ${\text{F}}_{nu}^{1}$ fibrosis.

#### 3.4.4. Threshold Selection

To evaluate the impact of using the optimal thresholds *D*^th^ and *R*^th^ for each configuration, Ac, Se, and FPR were computed again using the mean optimal thresholds value for all situations, reported in Tables 2, 3. The results varied only minimally. The global mean Ac decreased from 0.91 to 0.88, the global mean Se from 0.92 to 0.91 and the global mean FPR from 0.09 to 0.01.

### 3.5. Effects of Noise and Tissue-Electrode Distance

**Figure 10:**
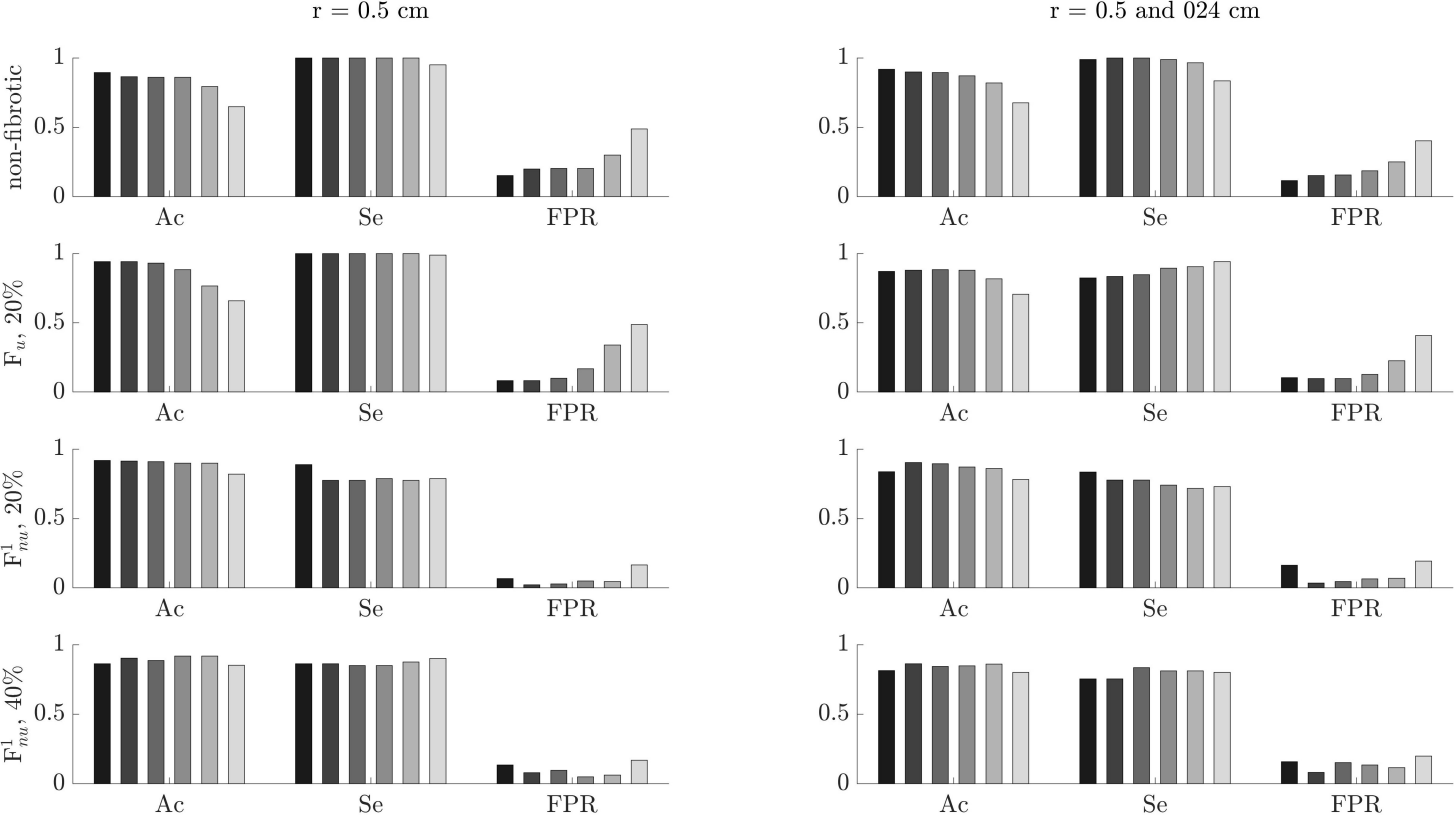
Accuracy (Ac), sensitivity (Se), and false positive rate (FPR) for different noise levels. The color code from dark to light gray is representative of no noise and noise with signal-to-noise ratio (SNR) values of 20, 15, 10, 5, and 0 dB.

The performance of the algorithm for the identification of ACh-release areas was tested on noisy signals with different SNR values of 0, 5, 10, 15, and 20 dB, corresponding to σ values of 279.2, 149.6, 89.7, 47.8, 26.9 μV, respectively. The results are displayed in Figure 10. For non-AF tissues and PxAF and PsAF tissues with F_*u*_, Ac and FPR values were highly decreased and increased, respectively, with the level of noise, however, still showing the Ac values of above 0.76 and FPR values below 0.29 when the SNR was 0 dB. On the other hand, Se was less dependent on noise and had values above 0.7 even for an SNR value of 0 dB. For PxAF and PsAF tissues with non-uniform diffuse fibrosis F_*nu*_, the performance of the algorithm was remarkably less dependent on the noise level. In the worst scenarios of SNR being 0 dB, the minimum Ac and Se values over all simulated cases with F_*nu*_ were 0.7 and of 0.63, respectively, while the maximum FPR value was 0.55.

**Figure 11:**
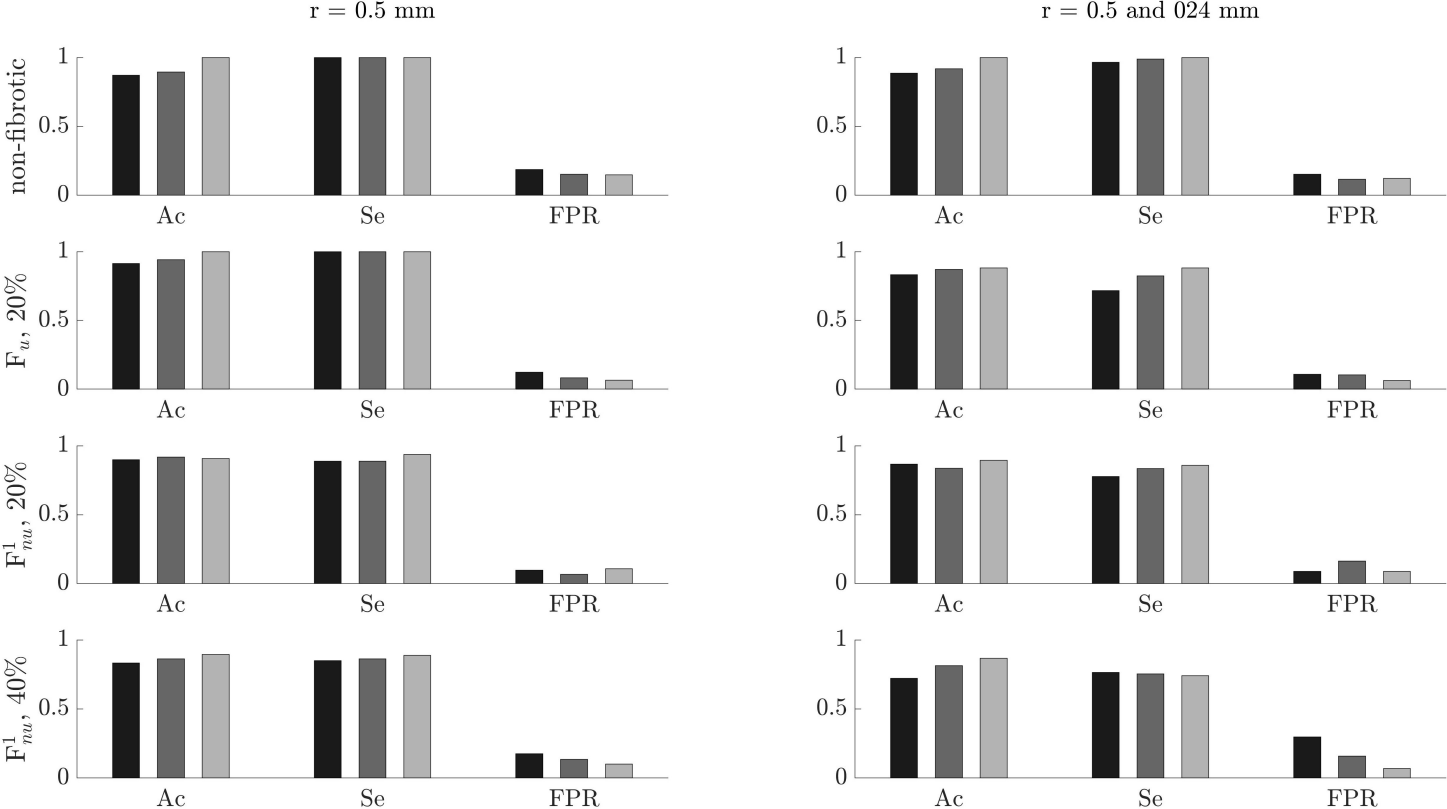
Accuracy (Ac), sensitivity (Se), and false positive rate (FPR) for different electrode-to-tissue distances. The color code from dark to light gray is representative of 2, 1, and 0.5 mm distances.

To test the effect of electrode-tissue distance, 3 different distances in the orthogonal direction z, namely 0.5, 1, and 2 mm, were used for EGM calculation in all the simulated cases. The performance of the algorithm as a function of the electrode-tissue distance is reported in Figure 11. Ac and Se modestly increased when the electrode came closer to the tissue, while FPR rate slightly increased or decreased with the electrode-tissue distance depending on the characteristics of the tissue.

### 3.6. Assessment of Model Dependence, Cell-To-Cell Variability, and Concomitant β-Adrenergic Stimulation

When using the Grandi model, although the time windows for repolarization analysis were different with respect to the ones obtained with the Courtemanche model, delineation could be successfully applied to the EGM waveforms. As in the Courtemanche cases, positive *R*_*i,j*_(*t*) waves were found in the ACh patches. A figure showing the corresponding results for the Grandi and Courtemanche model is presented in Supplementary Figure 11.

Including cell-to-cell variability in the Courtemanche model, as explained in the Materials and Methods section, led to similar results from a qualitative point of view, even if some quantitative differences could be observed. A comparison of the same case considering and not considering cell-to-cell variability can be found in Supplementary Figure 12.

Incorporating β-adrenergic stimulation into some atrial sites led to Iso regions being detected as non-ACh regions on the basis of the amplitude of the *R*_*i,j*_(*t*) waves. Based on these results, this method seems to be specifically meant to locate parasympathetic stimulated areas. A figure showing these results can be found in the Supplementary Figure 13.

## 4. Discussion

We developed a novel method to identify ACh release sites in the atrial myocardium based on the characteristics of EGM signals on a grid of electrodes. The method is based on evaluating the amplitude of the EGM repolarization wave and compare it with a threshold that is dependent on the presence or absence of fibrosis in the tissue portion beneath the EGM electrode position. An accompanying method for fibrosis detection is considered based on the amplitude of the EGM depolarization wave. The performance of this proposed method for identification of ACh release sites in the atrial myocardium was tested in simulated tissues representative of control (non-AF), PxAF, and PsAF. The simulated patterns of propagation resulted from pacing an entire edge of the tissue model at a fixed CL for all the cases, with no other AF patterns being simulated. We found that, in all cases, the algorithm was successful in identifying most of the simulated ACh release sites, both when there was no fibrosis in the tissue and when fibrosis was present in uniform diffuse or non-uniform diffuse forms at various degrees. In mean, accuracy and sensitivity values above 90% were achieved.

The role of the extrinsic and intrinsic CANS in modulating cardiac electrical behavior is well evidenced in the literature. Alterations in autonomic activity are documented to contribute to both initiation and maintenance of AF (Volders, 2010; Nishida et al., 2011; Oliveira et al., 2011; Shen et al., 2011; Arora, 2012; Ripplinger et al., 2016; Avazzadeh et al., 2020). Decreasing ANS innervation is shown to reduce the incidence of atrial arrhythmias (Chiou and Zipes, 1998; Schauerte et al., 2000; Pappone et al., 2004; Scanavacca et al., 2006). Specifically, regarding the intrinsic CANS, stimulation of GPs is reported to make them hyperactive and secrete excessive amounts of neurotransmsitters, which facilitates not only the initiation of AF but also its perpetuation. A study evaluating the intensity of vagal responses induced by endocardial high-frequency stimulation (HFS) of left atrial GPs highlights an increase in such responses in patients with AF compared with patients with non-AF, suggesting an abnormally increased GP activity in AF substrates (Iso Kazuki et al., 2019). Some studies have suggested that the extrinsic autonomic input to the heart exerts inhibitory control over GPs and, consequently, attenuation of this control allows GPs to become hyperactive (Stavrakis et al., 2015). On top of elevated neurotransmitter release, other possible explanations for GP hyperactivity have been reported (Iso Kazuki et al., 2019). One explanation is through its link to oxidative stress, which is strongly related to AF and can cause nerve injury (Shimano et al., 2009). This injury, in fact, can trigger the expression of nerve growth factors and neurotrophic factors in non-neuronal cells near the site of the lesion (Feng et al., 2002), which can lead to nerve sprouting. Another possible explanation is the increase in the density of the neural network, with an increased number of sympathetic and parasympathetic neurons, in association with GP-induced AF (Yu et al., 2014).

All the above effects related to GP stimulation involve both the sympathetic and parasympathetic divisions of the ANS. Cholinergic stimulation shortens the APD and leads to membrane hyperpolarization, while β-adrenergic stimulation can induce early afterdepolarizations, leading to rapid, triggered firing (Nattel et al., 2000; Patterson et al., 2006; Po et al., 2006; Iso Kazuki et al., 2019). Of the two, a predominant role of the parasympathetic division has been reported (Rysevaite et al., 2011; Krul et al., 2014). Here, we modeled the electrophysiology of 2D atrial tissues for non-AF, PxAF, and PsAF conditions, in which we incorporated circles of ACh release with different locations and/or sizes. These simulations confirmed that cholinergic stimulation of the atrial myocardium varies as a function of the atrial substrate, particularly depending on the amount and distribution of fibrosis. Also, while, in this study, we simulated the effects of 0.1 μM ACh dose in all cases and all ACh release sites, future studies could account for different ACh doses, possibly in relation to the number of available ACh receptors.

GP ablation as an adjunct to PV isolation has been shown to improve the outcomes of ablation procedures in patients with PxAF and PsAF (Po et al., 2009; Katritsis et al., 2013). In a canine study, where GPs are surgically removed, this technique is reported to acutely reduce the effects of vagosympathetic trunk stimulation on the atrial myocardium in an extensive way (Sakamoto et al., 2010). In situations where the activity of GPs facilitates the initiation and maintenance of AF, GP ablation could result in more beneficial than detrimental effects (Krul et al., 2014); however, there are still a several aspects related to GP ablation that deserve further investigation, including whether reinnervation occurs in the long-term after GP ablation and whether this could contribute to AF recurrence. In any case, if clinical trials with a large number of patients and long follow-up establish the efficacy of GP ablation, accurate location of GPs should be key and the proposed approach could find a place in the identification of GP location and extension. The methods currently available to locate GPs are the so-called functional approach, based on HFS (Lemery et al., 2006; Po et al., 2009), and the so-called anatomical approach, which uses anatomical mapping to identify the presumed GP locations (Pokushalov et al., 2009). Both methods, however, show limitations. On the one hand, the anatomical targeting of GP sites leaves the question open regarding the extent of GP area to be ablated, which could vary from one to another patient. On the other hand, GP sites identified by functional HFS may require several cardioversions following AF induction before testing for additional sites. Also, the equipment to perform HFS is not commonly available in all hospital facilities where ablation is performed and, importantly, studies have shown that GP ablation based on the functional approach may lead to higher AF recurrence than that based on the anatomical approach in patients with PxAF (Pokushalov et al., 2009; Krul et al., 2014; Lim and Kanagaratnam, 2015).

Considering that atrial EGM mapping is normally performed during ablation procedures, this study aims to find EGM features that serve to locate ACh release sites in the atrial myocardium. From the simulated atrial tissues, we computed EGMs on a high-density mesh of 16 × 16 electrodes. We started by characterizing changes in the amplitude of the EGM atrial repolarization wave under the presence of cholinergic stimulation, showing an increased amplitude in myocardial sites stimulated by ACh. This can be explained by a faster phase 3 of action potentials in cholinergically stimulated cells. When setting a threshold on the repolarization amplitude to identify ACh release sites, that threshold was found to be different according to the amount of fibrosis in the myocardium beneath the EGM electrode. Considering that there are regions in the atria, like the posterior wall of the LA, that are preferential locations for GPs and fibrosis (Yang et al., 2017; Benito et al., 2018), we used a method to distinguish fibrotic vs. non-fibrotic areas before applying this algorithm for the identification of ACh sites. There are only a few previous studies in the literature characterizing ACh effects on atrial EGMs. In Lellouche et al. (2007), EGM patterns from patients with PxAF are compared with *in silico* simulated EGMs. ACh release sites are associated with fractionated EGMs, being the number of deflections the first predictor of cholinergic response. In Vigmond et al. (2005) and Vigmond et al. (2009), high density electrical recordings were acquired from dogs under vagal stimulation and computational simulations are performed. I increased amplitude of the atrial repolarization wave was found in and around the ACh islands. In that study, however, the interaction between ACh release sites and different amounts and distributions of fibrosis was not investigated. This study confirms the findings from that study regarding ACh effects in non-AF tissues and further extends the results to PxAF and PsAF substrates in the presence of electrical and/or structural remodeling, highlighting the need to first identify fibrotic areas. To identify those areas, we used the amplitude of the EGM depolarization wave, as commonly performed in clinical practice (Rolf et al., 2014; Blandino et al., 2017; Nairn et al., 2020). When the amount of fibrosis was high (40%), the detection of fibrotic areas was successful. This performance was, however, reduced under lower levels of fibrosis (20%) if this was distributed in patches, making the error subsequently propagate to identification of ACh sites. Other strategies for fibrosis detection based on bipolar EGM amplitude or using shape-based methods (Riccio et al., 2020) could improve the performance in those specific cases.

To identify ACh areas based on the amplitude of the repolarization waves, we preprocessed the EGM signals by application of a 2 Hz highpass filter, which is a possibility with the NavX system (Endocardial Solutions, St. Jude Medical, Inc., St. Paul, MN, USA), but not with others, the Carto^Ⓡ^ system (Biosense Webster, Baldwin Park, CA, USA), where the lowest cutoff frequency is around 5 Hz and highpass filtering the signal using that cutoff frequency could lead to *R*_*i,j*_(*t*) wave cancellation. This method should, thus, be adjusted depending on the characteristics of the system. Regarding performance, we can conclude that this algorithm successfully identifies the ACh release sites, particularly in tissues with no fibrosis or with diffusive fibrosis and uniform dimensions of the ACh patches. In the case of tissues with non-uniform diffuse fibrosis, the smaller ACh release sites can only be partially identified, or in some cases not identified, if they lie in fibrotic regions. Since in the clinical setting, it is likely that only the major GPs are of interest for ablation procedures, the strategy is expected to work fine. Indeed, our method generally produces very good results for the largest ACh release sites, providing information not only on their location but also on their extension. It should be noted that the method was tested on both PxAF and PsAF substrates. Some studies show that the improvement in the outcome of ablation procedures is mainly observed in PxAF (Pokushalov et al., 2009; Chao et al., 2012). When ablation includes GPs, this could be explained according to the hypothesis that autonomic hyperactivity plays a more predominant role in the early stages of AF development and that its relevance decreases with the progression of the disease and the consequent structural remodeling (Stavrakis et al., 2015). Independently of the amount of GP hyperactivity, the method proposed, in this study, can locate ACh release sites with high performance, with mean Ac and Se, over all, simulated cases being above 0.91.

### 4.1. Limitations

Some limitations of this study should be acknowledged to provide direction for further work. We investigated 2D atrial tissue sheets with different fibrosis distributions and ACh-release areas. Although we did not include regional electrophysiological heterogeneities in the tissues, we confirmed that introducing cell-to-cell variability in electrical properties led to the same qualitative conclusions. Further studies could implement 3D atrial geometries with more realistic GP distributions, accounting for its structural complexity and incorporating many of the regional electrical heterogeneities present in the intact organ. In addition different stimulation sites and protocols could be tested to assess the impact of other activation patterns on the performance of the proposed methods.

The differentiation between PxAF and PsAF is challenging. Current clinical AF classification (paroxysmal, persistent, long-term persistent, and permanent) is based on the duration of AF episodes and the form of termination (January Craig T. et al., 2014). AF is considered to be a progressive disease, starting from short and infrequent episodes to longer and more frequent ones. This progression is overall shown to be accompanied by alterations in a myocardial substrate. The hallmark of structural remodeling in AF is the increase in the amount of fibrosis, which is, in general, significantly higher in with AF compared with patients in sinus rhythm and in PsAF compared with PxAF (Platonov et al., 2011). However, different studies have highlighted large inter-individual variability in the fibrotic load in PxAF and PsAF, reporting individual cases of patients with PxAF showing massive fibrosis as well as patients with PsAF showing mild fibrosis (Boldt et al., 2004; Oakes et al., 2009; Platonov et al., 2011; Teh et al., 2012). These models reflect a simplified categorization of AF, with fibrotic load increasing with AF progression. As such, they should serve as a proof of concept of the feasibility of the proposed method for different AF substrates.

Atrial structural remodeling in AF can manifest as enlargement of the atrial chamber, cardiomyocyte hypertrophy, increased mismatch between the orientations of epicardial and endocardial myofibers, changes in atrial wall thickness and, importantly, increased content of fibrotic or connective tissue (Schotten et al., 2011; Wyse et al., 2014; Heijman et al., 2016). Fibrosis remodeling is a multiscale process involving from subcellular to tissue levels. It has been associated with gap junction remodeling (Kostin et al., 2002; Burstein Brett et al., 2009), fibroblast proliferation (Rohr, 2009; Yue et al., 2011) and excess collagen deposition (Burstein and Nattel, 2008; Yue et al., 2011), all interfering with cardiac electrical propagation and slowing conduction. Computational models of fibrotic atria have accounted for these remodeling aspects, either separately or in combination; however, due to the need for further experimental characterization from human tissue, additional work should help to shed light on how to best model atrial fibrosis in humans (Smaill Bruce H. et al., 2013). In this study, we considered a combination of gap junction remodeling, modeled through tissue conductance reduction in fibrotic regions, as well as fibroblast proliferation. The latter is modeled by replacing myocytes with fibroblasts and using a fibroblast ionic model (Andrew MacCannell et al., 2007), as in previous studies (McDowell et al., 2013). Importantly, fibroblasts can exert electrophysiological influences on neighboring myocytes (Pedrotty et al., 2009), shortening APD, slowing conduction and lowering excitability (Trayanova, 2014). In these models, we did not consider the increase in collagen content, which is usually modeled as non-conductive obstacles in the tissue; however considering that increased collagen content in the interstitial spaces between fibers has been found, in general, not to affect longitudinal conduction (Burstein Brett et al., 2009; McDowell et al., 2012, 2013). Since we simulated a planar wavefront, this would not be expected to alter these results.

When simulating electrical remodeling in PsAF, we also considered the possibility to add I_*K*1_ current remodeling, as several studies have reported an increase in this current in PsAF by a factor of two or more (Bosch et al., 1999; Dobrev et al., 2001). Simulation of such an I_*K*1_ increase in the Courtemanche model required reducing the ACh concentration to 0.01 μM for an AP to be elicited. Therefore, we considered a 50% of I_*K*1_ increase as observed in Shim et al. (2017), which allowed considering an ACh concentration of 0.05 μM. The APs obtained from single-cell simulations are reported in the Supplementary Figure 14. The results in terms of *R*_*i,j*_(*t*) after including I_*K*1_ remodeling are qualitatively similar to the ones presented without including I_*K*1_ remodeling in PsAF. It should be noted that, since ACh concentration was reduced, this led to a reduction in the amplitude of the repolarization waves', which occurs with and without I_*K*1_ remodeling, the latter occurs both for PsAF and PxAF cases. Simulation results showing these effects are reported in the Supplementary Figure 15. Other currents like I_*K, ACh*_ have additionally been reported to possibly present PsAF-associated remodeling (Dobrev et al., 2001). Future studies could include modeling of I_*K, ACh*_ changes in PsAF based on available experimental evidence.

To find the optimal values of depolarization and repolarization amplitude thresholds for the identification of fibrotic and ACh regions, we used a statistical ROC curve analysis. For this method to be applicable in clinical practice, another set of data would be required to build the ROC curves, as in the set of data under analysis, the Se and Sp values would not be available *a priori*. To evaluate the impact of the thresholds on the method performance, we alternatively computed Ac, Se, and FPR using the mean values of depolarization and repolarization amplitudes in the tissue being analyzed. We found only minimal differences, thus confirming that the performance was not degraded. This proves the robustness of this method using mean thresholds even if only in the simulation framework. In the clinical setting, one possibility to retrieve data to build ROC curves for the analysis of EGMs from patients would be to collect those EGMs while performing HFS location of GP. This data could be analyzed offline to identify the optimal thresholds and compare the performance of the method using mean amplitude thresholds against the one using optimal thresholds. Upon confirmation of the suitability of using mean thresholds, these could be later used in real time. We aim at undertaking future studies extending the present one, where we will test our method on EGM signals from patients collected as just described, over which will apply the procedure developed.

Finally, detection of the atrial repolarization wave in EGMs could be difficult due to contamination related to ventricular depolarization activity; however, some works have already investigated the feasibility of the identification and analysis of unipolar atrial repolarization waves of EGM. Jousset et al. investigated principal component analysis of intracardiac unipolar EGMs to subtract ventricular activity (Jousset, 2011). Other studies by the same group (Jousset et al., 2012; Monigatti-Tenkorang et al., 2014) characterized atrial repolarization in sheep. Jousset et al. considered two sets of animals: one with AV block to prevent far-field ventricular depolarization impinging on the preceding atrial repolarization, and the other one without AV block. A method for ventricular activity cancellation was used in the AV block group. In any case, atrial repolarization alternans were detected from unipolar EGMs in all sheep. The apex of the atrial repolarization wave was evaluated, which could serve as a basis to support the feasibility of measuring atrial repolarization amplitude, as proposed in this study. In another study, atrial repolarization waves were characterized using high resolution atrial bipolar EGMs, and the effects of vagal nerve stimulation on repolarization duration were assessed (Verrier et al., 2016). Future studies extending the present work could investigate atrial repolarization measured from bipolar EGMs for the identification of ACh release sites.

## 5. Conclusions

This study develops a method to locate atrial ACh release sites based on the analysis of the repolarization phase amplitude of EGM signals from a grid of electrodes. The method is tested in simulated non-AF, PxAF, and PsAF tissues with different sizes and locations of ACh and fibrosis areas, in which propagation patterns were simulated by pacing an entire edge of the tissue model at a constant CL. In all simulated cases, the method is able to identify ACh sites with an accuracy above 0.8, being the mean larger than 0.91. The method is robust against noise and works well with various EGM electrode distances. Despite simplification in the *in silico* modeling of non-AF, PxAF, and PsAF tissues, these results could serve as a proof of concept for the feasibility of unveiling ACh sites from atrial electroanatomical mapping during ablation interventions.

## Data Availability Statement

The raw data supporting the conclusions of this article will be made available by the authors, without undue reservation.

## Author Contributions

CC, CS, PL, and EP designed the study. CC, CS, KM, PL, and EP analyzed the results. CC drafted the manuscript, developed the software, and performed the required computations. CC and KM performed the electrophysiological simulations. KM contributed with technical details. EP, PL, and CS supervised and formalized the project and were responsible for overseeing the research and providing critical insight and recommendations regarding the focus, structure and content of the manuscript. All authors critically revised the manuscript and approved the submitted version.

## Conflict of Interest

The authors declare that the research was conducted in the absence of any commercial or financial relationships that could be construed as a potential conflict of interest.

## Publisher's Note

All claims expressed in this article are solely those of the authors and do not necessarily represent those of their affiliated organizations, or those of the publisher, the editors and the reviewers. Any product that may be evaluated in this article, or claim that may be made by its manufacturer, is not guaranteed or endorsed by the publisher.

## Supplementary Material

Supplementary Figure 1 EGM analysis for a non-AF tissue. **(Top)** EGMs aligned with respect to the time correspondent to the maximum slope of the depolarization wave (marked as 0 in the x-axis). The vertical dashed lines delimit the time window TW for the *R*_*i,j*_(t) repolarization signals. **(Bottom)** Atrial repolarization waves, *R*_*i,j*_(t), with dots indicating the maximum absolute value *R*${}_{i,j}^{A}$ of the waves within TW. The horizontal dotted lines represent the optimal threshold *R*^th^ found by Se/Sp analysis.

Supplementary Figure 2 EGM analysis for the uniform diffusive fibrosis PxAF tissue (F_*u*_ 20%). The figure is structured as Supplementary Figure 1.

Supplementary Figure 3 EGM analysis for the uniform diffusive fibrosis PsAF cases (F_*u*_ 40%). The figure is structured as Supplementary Figure 1.

Supplementary Figure 4 EGM analysis for a PxAF tissue with 20% patchy type 1 fibrosis (type 1 patchy fibrosis is represented in Figure 1E) in the main manuscript) (${\text{F}}_{\text{nu}}^{1}$ 20%). **(Top)** EGMs aligned with respect to the time correspondent to the maximum slope of the depolarization wave (marked as 0 in the x-axis). The vertical dashed lines delimit the time window TW for the *R*_*i,j*_(t) repolarization signals. **(Bottom)** Atrial repolarization waves, *R*_*i,j*_(t), with dots indicating the maximum absolute value *R*${}_{i,j}^{A}$ of the waves within TW. The horizontal dotted lines represent the optimal threshold *R*^*th*^ found by Se/Sp analysis.

Supplementary Figure 5 EGM analysis for a PxAF tissue with 20% patchy type 2 fibrosis (fibrosis geometry reported in Figure 1F) in the main manuscript) (${\text{F}}_{\text{nu}}^{2}$, 20%). The figure is structured as Supplementary Figure 4.

Supplementary Figure 6 EGM analysis for a PsAF tissue with 40% patchy type 1 fibrosis (fibrosis geometry reported in Figure 1E) in the paper) (${\text{F}}_{nu}^{1}$, 40%). The figure is structured as Supplementary Figure 4.

Supplementary Figure 7 EGM analysis for a PsAF tissue with 40% patchy type 2 fibrosis (fibrosis geometry reported in Figure 1F) in the main manuscript) (${\text{F}}_{nu}^{2}$, 40%). The figure is structured as Supplementary Figure 4.

Supplementary Figure 8 Results of the algorithm for detection of ACh release sites. Each electrode is assigned with non-ACh, ACh, non-ACh + fibro, or ACh + fibro on the basis of EGM analysis. The color code is the same as in Supplementary Figure 1.

Supplementary Figure 9 Results of the algorithm for detection of all the PxAF cases. Each electrode is assigned with non-ACh, ACh, non-ACh + fibro, or ACh + fibro on the basis of EGM analysis. The color code is the same as in Supplementary Figures 2–7.

Supplementary Figure 10 Results of the algorithm for detection of all the PsAF cases. Each electrode is assigned with non-ACh, ACh, non-ACh + fibro, or ACh + fibro on the basis of EGM analysis. The color code is the same as in Supplementary Figures 2–7.

Supplementary Figure 11 **(A)** 2D model of a non-AF tissue with ACh release sites shown in black and EGM electrodes in red and blue. Cell electrophysiology is simulated using the Grandi model. **(B,D)** APs/EGMs recorded in the (i,j) points represented in **(A)**. The thicker lines correspond to the points represented with big squares in the tissue. **(C)** represents the EGMs aligned with respect to the time correspondent to the maximum slope of the depolarization wave (marked as *t* = 0 in the x-axis). The vertical dashed lines delimit the time window TW for the *R*_*i,j*_(t) repolarization signals. The dots indicate the maximum absolute value, *R*${}_{i,j}^{A}$(t), of the waves within TW.

Supplementary Figure 12 EGM analysis for a non-AF tissue. **(A)** represents the uniform cells case while **(B)** represents the case which considers cell to cell variability. **(Top)** EGMs aligned with respect to the time correspondent to the maximum slope of the depolarization wave (marked as 0 in the x-axis). The vertical dashed lines delimit the time window TW for the *R*_*i,j*_(t) repolarization signals. **(Bottom)** atrial repolarization waves, *R*_*i,j*_(t), with dots indicating the maximum absolute value, *R*${}_{i,j}^{A}$, of the waves within TW. The horizontal dotted lines represent the optimal threshold *R*^*th*^ found by Se/Sp analysis.

Supplementary Figure 13 **(A)** 2D model of a non-AF tissue with ACh release sites shown in black, β-adrenergically stimulated sites in white and EGM electrodes in red, blue and green. **(B)** EGMs aligned with respect to the time correspondent to the maximum slope of the depolarization wave (marked as 0 in the x-axis). The vertical dashed lines delimit the time window TW for the *R*_*i,j*_(t) repolarization signals. **(C)** Atrial repolarization waves, *R*_*i,j*_(t), with dots indicating the maximum absolute value, *R*${}_{i,j}^{A}$, of the waves within TW.

Supplementary Figure 14 Action potentials obtained from single cell simulations pacing the cell at 1 Hz for 60 s. The continuous lines represent AP when ACh is not added, while the dashed lines represent the same cases with the addition of 0.05 μM ACh. The black lines represent the formulation of PsAF adopted in the manuscript, while the red lines represent the results after including 50% increase in I_*K*1_.

Supplementary Figure 15 EGM analysis for the case with 0.5 cm ACh patches and 20% or 40% ${\text{F}}_{nu}^{1}$. **(A)** represents PsAF tissues without I_*K*1_ remodeling and ACh = 0.1 μM. **(B)** represents PsAF tissues with I_*K*1_ remodeling and ACh = 0.05 μM. **(C)** shows the PxAF case with ACh = 0.1 μM. **(D)** corresponds to the PxAF case with ACh = 0.05 μM. **(Top)** EGMs aligned with respect to the time correspondent to the maximum slope of the depolarization wave (marked as *t* = 0 in the x-axis). The vertical dashed lines delimit the time window TW for the *R*_*i,j*_(t) repolarization signals. **(Bottom)** atrial repolarization waves, *R*_*i,j*_(t), with dots indicating the maximum absolute value, *R*${}_{i,j}^{A}$, of the waves within TW. The horizontal dotted lines represent the optimal threshold *R*^*th*^ found by Se/Sp analysis.
