# Supplementary material for: Location of Parasympathetic Innervation Regions From Electrograms to Guide Atrial Fibrillation Ablation Therapy: An in silico Modeling Study
Source: Front Physiol. 2021 Aug 11;12:674197. doi: 10.3389/fphys.2021.674197 (PMC8385640; doi:10.3389/fphys.2021.674197)
Supplement: Supplementary Figure 1 — EGM analysis for a non-AF tissue. (Top) EGMs aligned with respect to the time correspondent to the maximum slope of the depolarization wave (marked as 0 in the x-axis). The vertical dashed lines delimit the time window TW for the Ri,j(t) repolarization signals. (Bottom) Atrial repolarization waves, Ri,j(t), with dots indicating the maximum absolute value Ri,jA of the waves within TW. The horizontal dotted lines represent the optimal threshold Rth found by Se/Sp analysis. [file Data_Sheet_1.pdf]

Supplementary Material

Equation for EGM computation in the discretized form:

$$\phi_e(\mathbf{r}',t) = \sum_{i=1}^{N-1}\sum_{j=1}^{N-1}\left[-\left(\frac{\Delta_x V_m(\mathbf{r}_{i,j},t)}{\Delta x}, \frac{\Delta_y V_m(\mathbf{r}_{i,j},t)}{\Delta y}\right)\cdot\left(\frac{\Delta_x \frac{1}{d(\mathbf{r}_{i,j},\mathbf{r}')}}{\Delta x}, \frac{\Delta_y \frac{1}{d(\mathbf{r}_{i,j},\mathbf{r}')}}{\Delta y}\right)\right]\Delta x \Delta y \quad \text{(S1)}$$

where $\mathbf{r}_{i,j} = \begin{bmatrix} x_i & y_j & z \end{bmatrix}$, $\mathbf{r}_{i+1,j} = \begin{bmatrix} x_{i+1} & y_j & z \end{bmatrix}$ and $\mathbf{r}_{i,j+1} = \begin{bmatrix} x_i & y_{j+1} & z \end{bmatrix}$ are the coordinate vectors, in the Cartesian system, of points in the 2D tissue. The distance function d is defined as:

$$d(\mathbf{r},\mathbf{r}') = ||\mathbf{r}-\mathbf{r}'||_2 \quad \text{(S2)}$$

where $\mathbf{r} = \begin{bmatrix} x & y & z \end{bmatrix}$ and $\mathbf{r}' = \begin{bmatrix} x' & y' & z' \end{bmatrix}$ are the coordinate vectors for a 2D tissue point and for the electrode, respectively. N is the number of nodes in the x and y directions, both equal to $N = 251$, and $\Delta x = \Delta y = 0.02$ cm. Finally, $\Delta_x V_m(\mathbf{r}_{i,j},t) = (V_m(\mathbf{r}_{i+1,j},t) - V_m(\mathbf{r}_{i,j},t))$, $\Delta_y V_m(\mathbf{r}_{i,j},t) = (V_m(\mathbf{r}_{i,j+1},t) - V_m(\mathbf{r}_{i,j},t))$, $\Delta_x \frac{1}{d(\mathbf{r}_{i,j},\mathbf{r}')} = (\frac{1}{d(\mathbf{r}_{i+1,j},\mathbf{r}')} - \frac{1}{d(\mathbf{r}_{i,j},\mathbf{r}')})$ and $\Delta_y \frac{1}{d(\mathbf{r}_{i,j},\mathbf{r}')} = (\frac{1}{d(\mathbf{r}_{i,j+1},\mathbf{r}')} - \frac{1}{d(\mathbf{r}_{i,j},\mathbf{r}')})$.

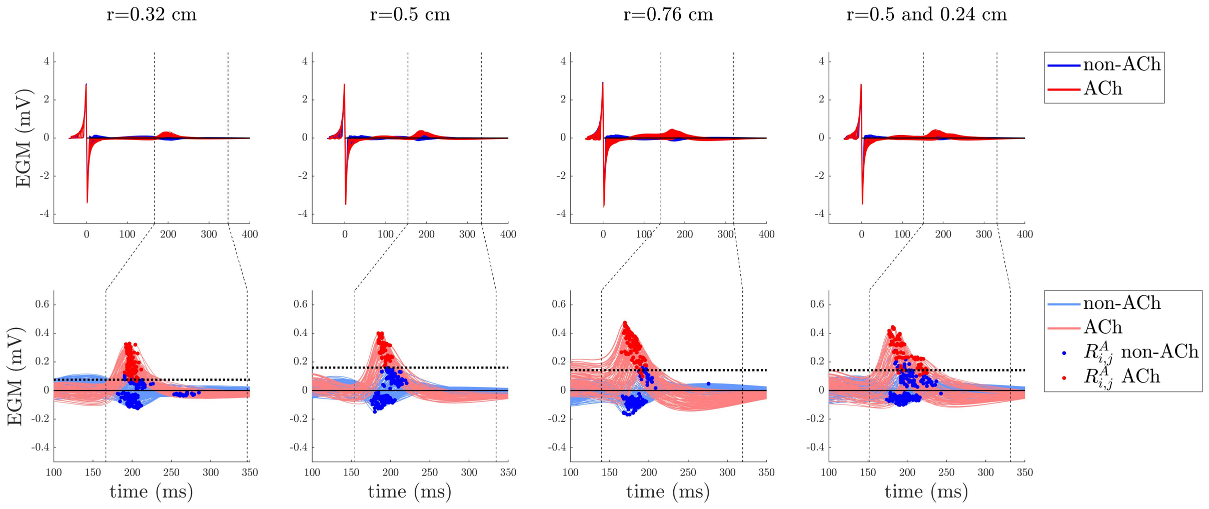

Figure S1: EGM analysis for a non-AF tissue. Top row: EGMs aligned with respect to the time correspondent to the maximum slope of the depolarization wave (marked as 0 in the x-axis). The vertical dashed lines delimit the time window TW for the $R_{i,j}(t)$ repolarization signals. Bottom row: atrial repolarization waves, $R_{i,j}(t)$, with dots indicating the maximum absolute value $R^A_{i,j}$ of the waves within TW. The horizontal dotted lines represent the optimal threshold R^{th} found by Se/Sp analysis.

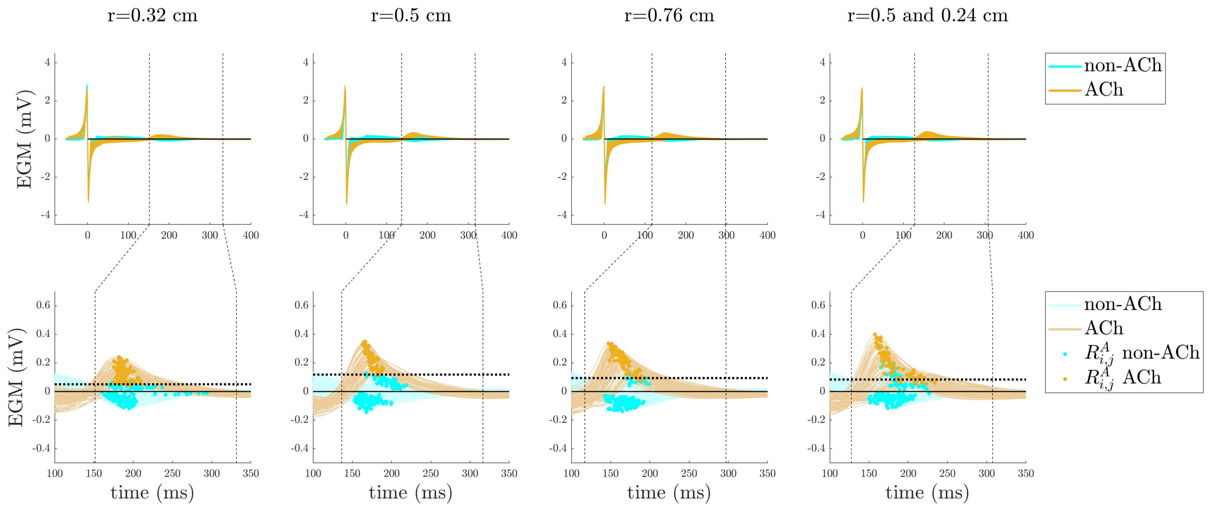

Figure S2: EGM analysis for the uniform diffusive fibrosis PxAF tissue (F_u 20%). The figure is structured as Fig. S1.

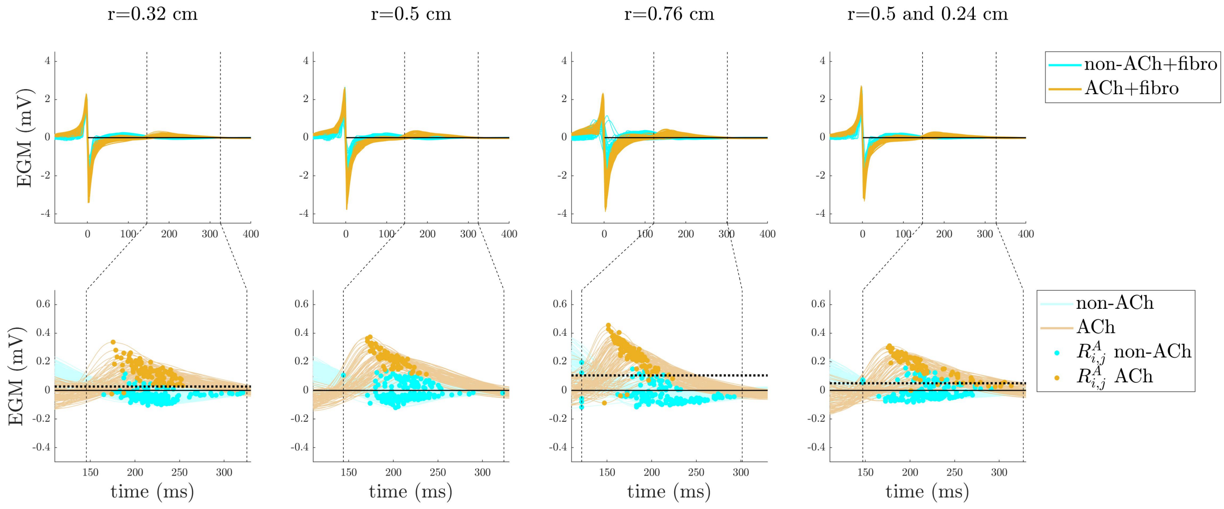

Figure S3: EGM analysis for the uniform diffusive fibrosis PsAF cases (F_u 40%). The figure is structured as Fig. S1.

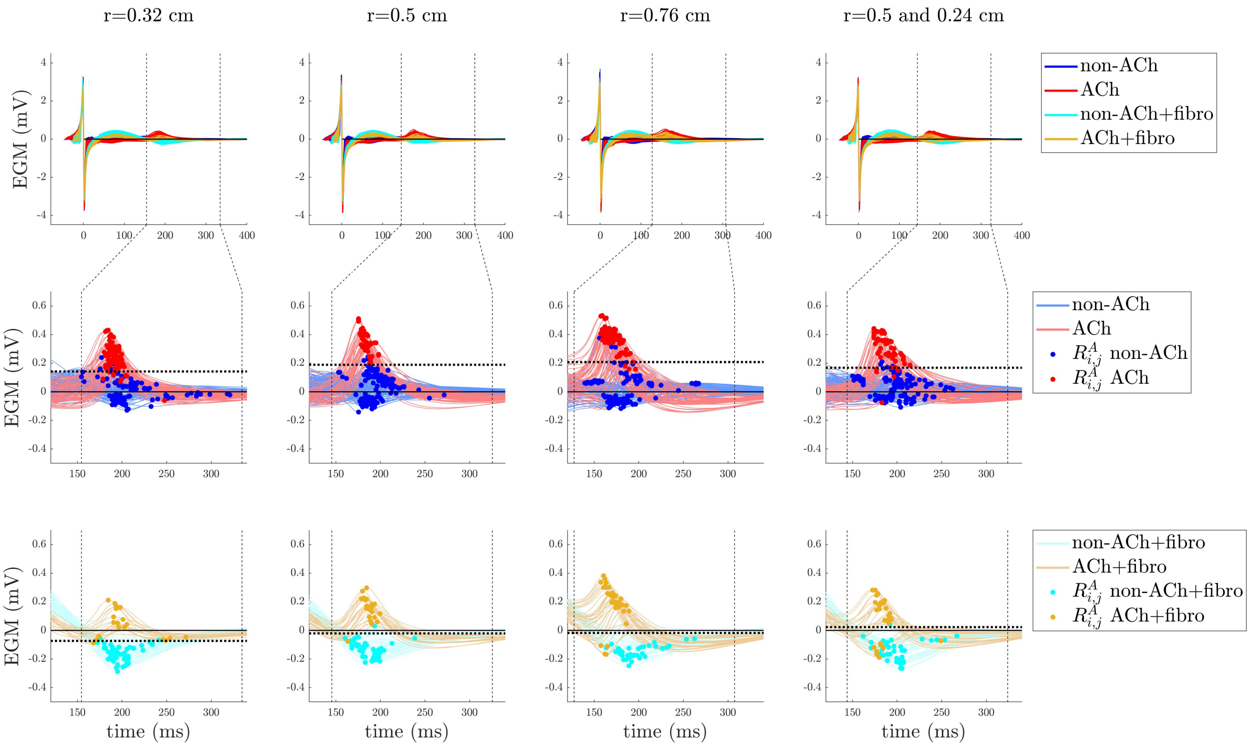

Figure S4: EGM analysis for a PxAF tissue with 20% patchy type 1 fibrosis (type 1 patchy fibrosis is represented in Fig. 1E) in the main manuscript) (F^1_{nu} 20%). Top row: EGMs aligned with respect to the time correspondent to the maximum slope of the depolarization wave (marked as 0 in the x-axis). The vertical dashed lines delimit the time window TW for the $R_{i,j}(t)$ repolarization signals. Bottom row: atrial repolarization waves, $R_{i,j}(t)$, with dots indicating the maximum absolute value $R^A_{i,j}$ of the waves within TW. The horizontal dotted lines represent the optimal threshold R^{th} found by Se/Sp analysis.

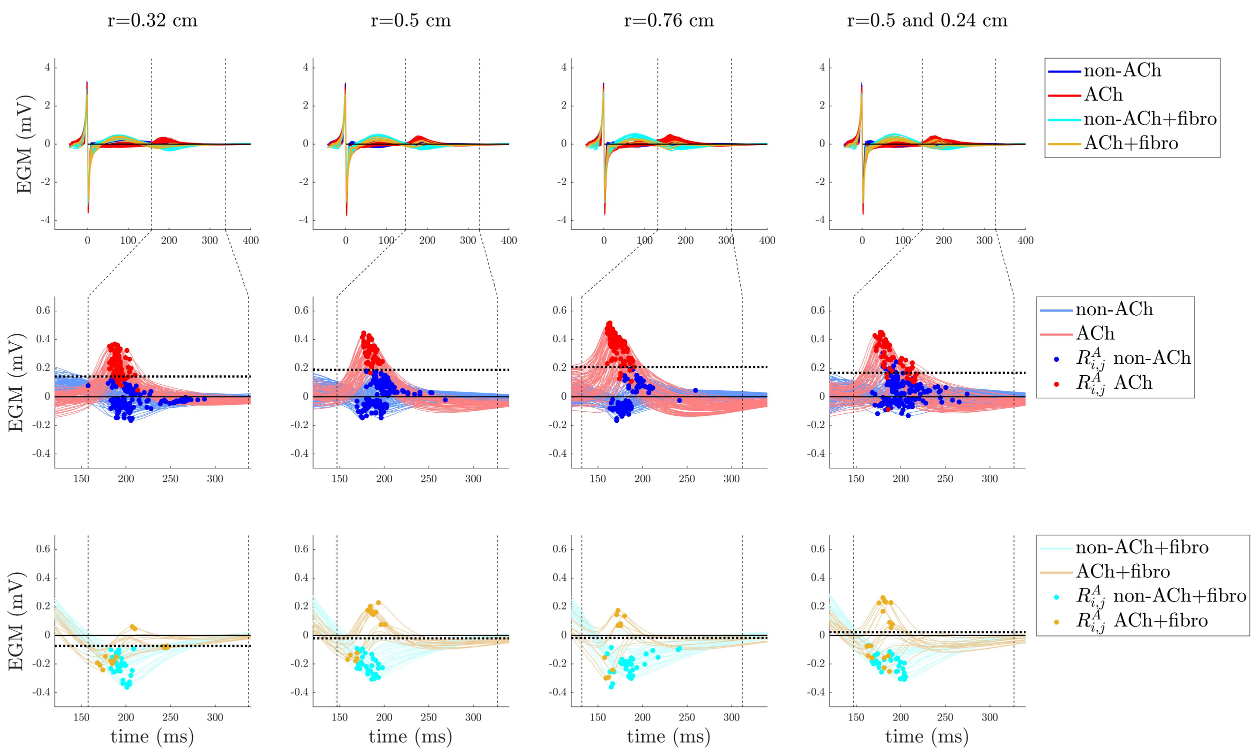

Figure S5: EGM analysis for a PxAF tissue with 20% patchy type 2 fibrosis (fibrosis geometry reported in Fig. 1F) in the main manuscript) (F^2_{nu}, 20%). The figure is structured as Fig. S4.

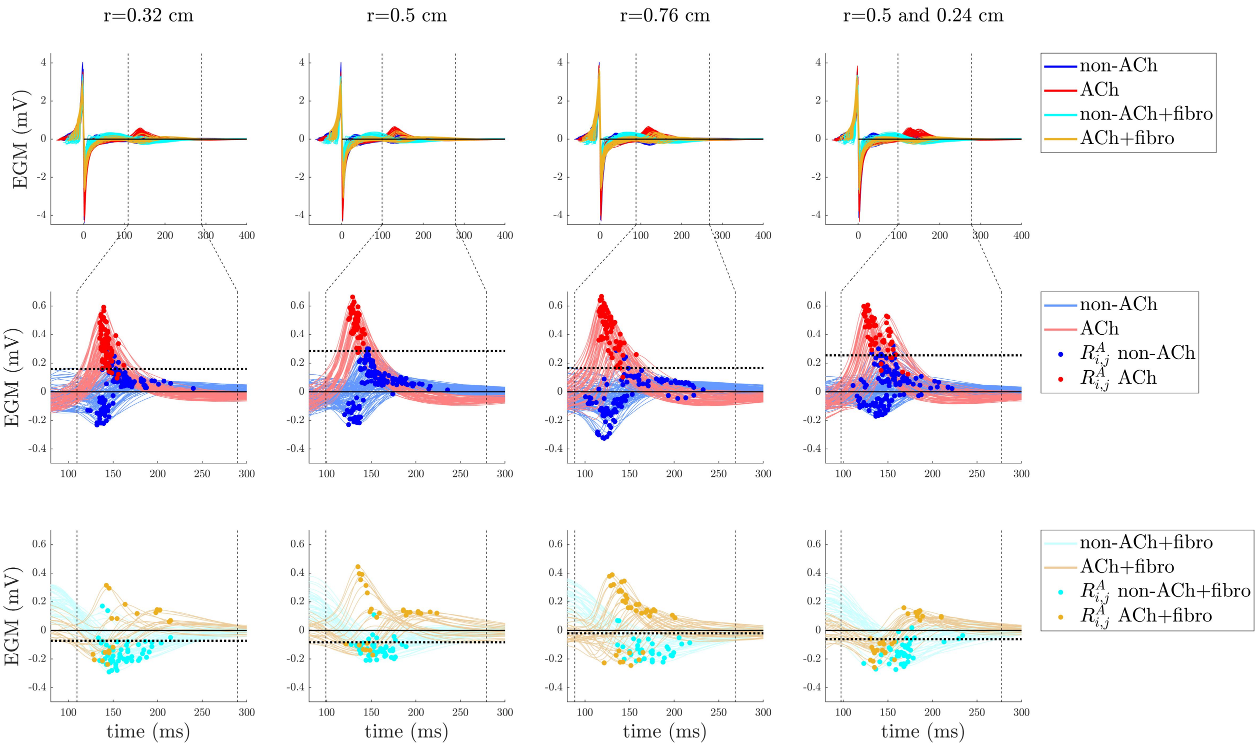

Figure S6: EGM analysis for a PsAF tissue with 40% patchy type 1 fibrosis (fibrosis geometry reported in Fig. 1E) in the paper) (F^1_{nu}, 40%). The figure is structured as Fig. S4.

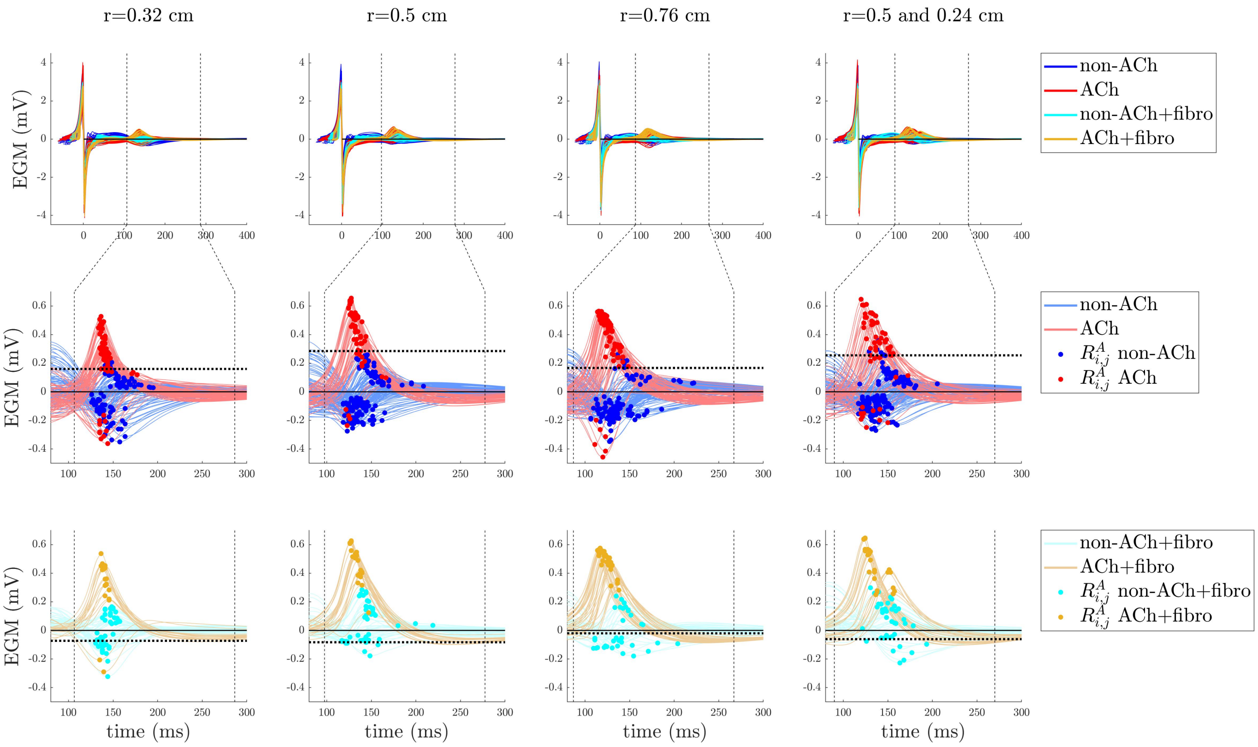

Figure S7: EGM analysis for a PsAF tissue with 40% patchy type 2 fibrosis (fibrosis geometry reported in Fig. 1F) in the main manuscript) (F^2_{nu}, 40%). The figure is structured as Fig. S4.

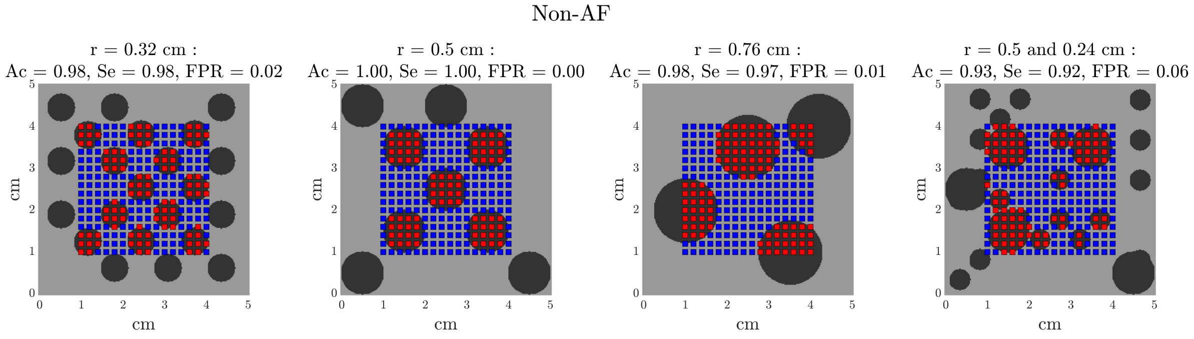

Figure S8: Results of the algorithm for detection of ACh release sites. Each electrode is assigned with non-ACh, ACh, non-ACh + fibro or ACh + fibro on the basis of EGM analysis. The color code is the same as in Fig. S1.

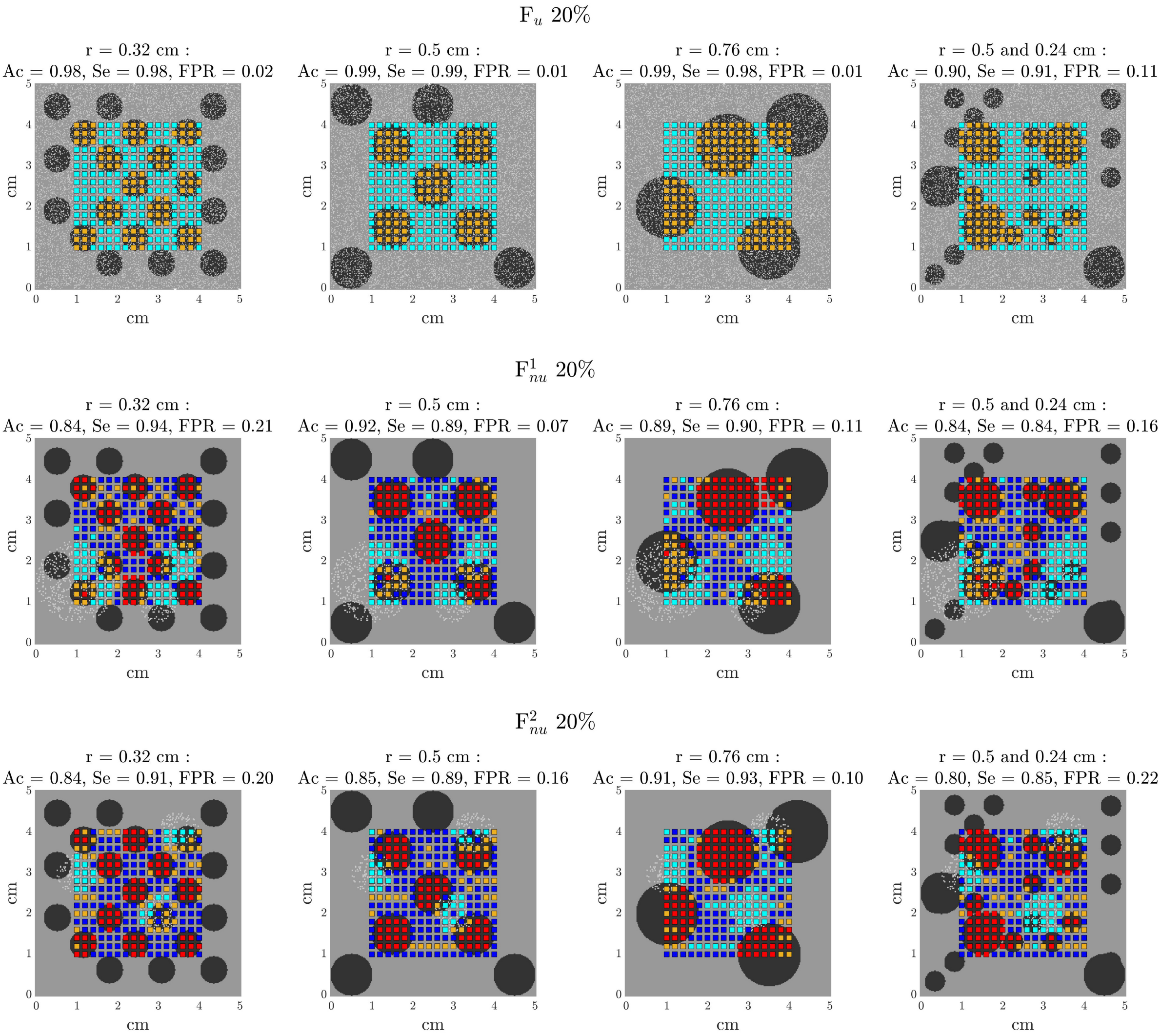

Figure S9: Results of the algorithm for detection of all the PxAF cases. Each electrode is assigned with non-ACh, ACh, non-ACh + fibro or ACh + fibro on the basis of EGM analysis. The color code is the same as in Fig. S2 to Fig. S7.

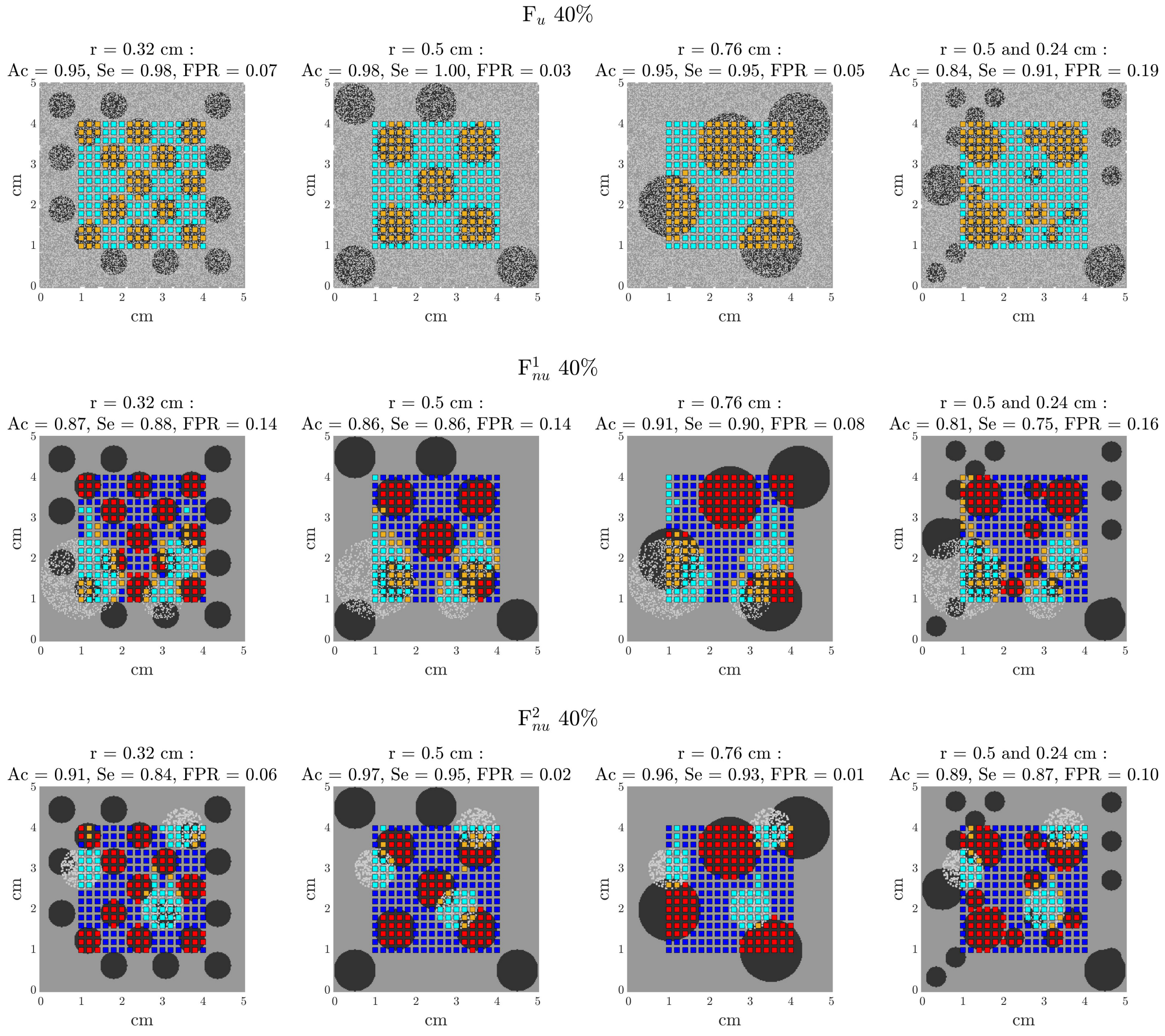

Figure S10: Results of the algorithm for detection of all the PsAF cases. Each electrode is assigned with non-ACh, ACh, non-ACh + fibro or ACh + fibro on the basis of EGM analysis. The color code is the same as in Fig. S2 to Fig. S7.

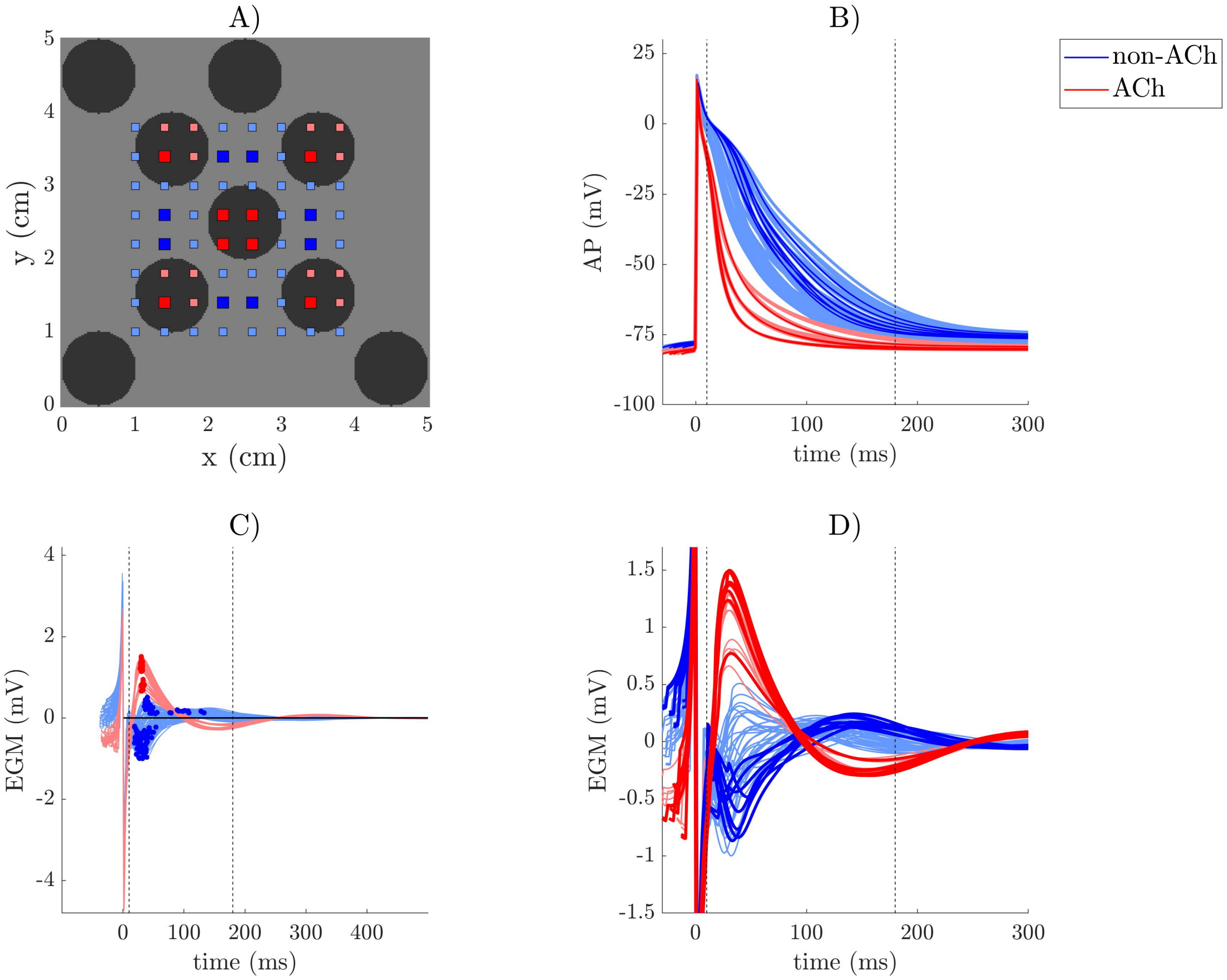

Figure S11: A) 2D model of a non-AF tissue with ACh release sites shown in black and EGM electrodes in red and blue. Cell electrophysiology is simulated using the Grandi model. B)/D) APs/EGMs recorded in the (i, j) points represented in panel A). The thicker lines correspond to the points represented with big squares in the tissue. Panel C) represents the EGMs aligned with respect to the time correspondent to the maximum slope of the depolarization wave (marked as $t = 0$ in the x-axis). The vertical dashed lines delimit the time window TW for the $R_{i,j}(t)$ repolarization signals. The dots indicate the maximum absolute value, $R^A_{i,j}$, of the waves within TW.

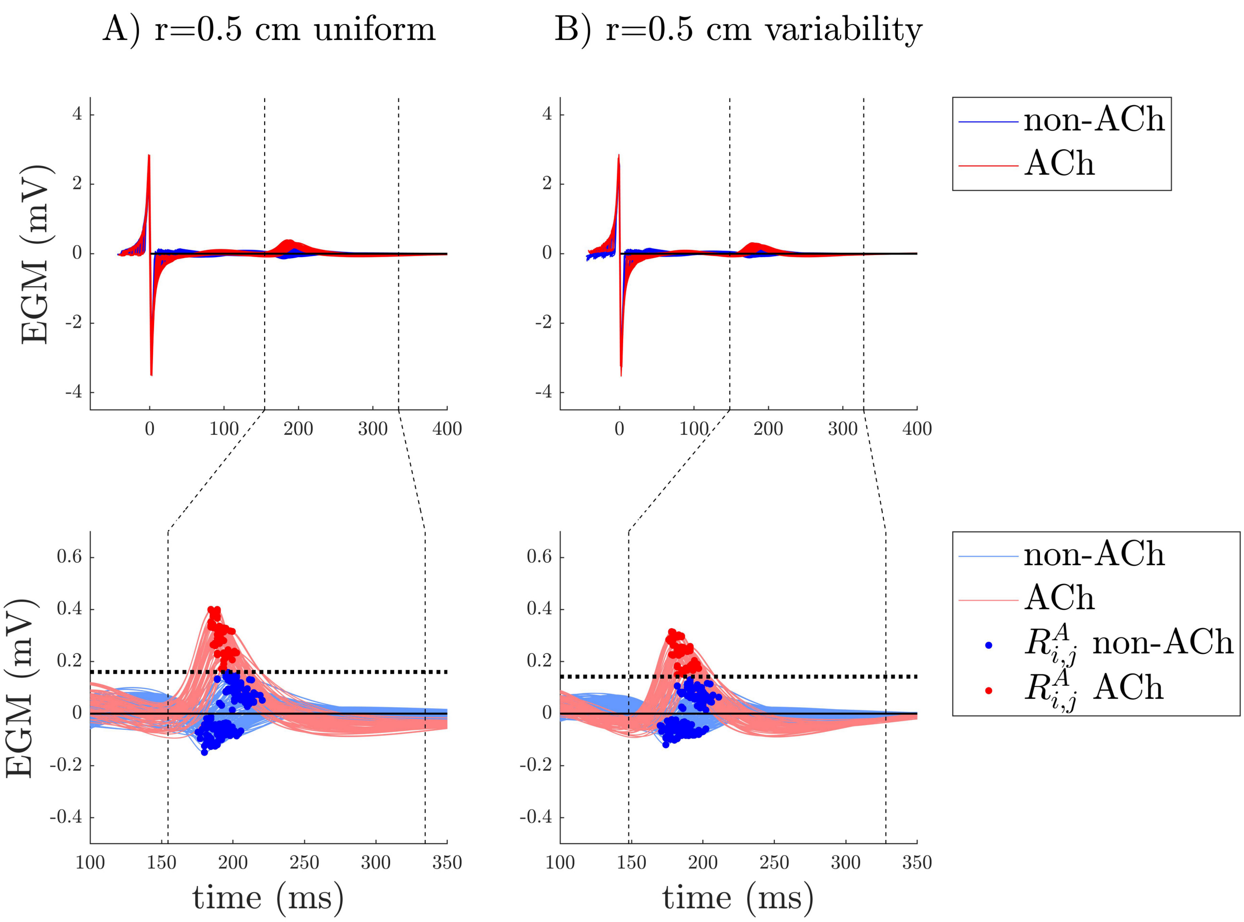

Figure S12: EGM analysis for a non-AF tissue. A) represents the uniform cells case while B) represents the case which considers cell to cell variabilty . Top row: EGMs aligned with respect to the time correspondent to the maximum slope of the depolarization wave (marked as 0 in the x-axis). The vertical dashed lines delimit the time window TW for the $R_{i,j}(t)$ repolarization signals. Bottom row: atrial repolarization waves, $R_{i,j}(t)$, with dots indicating the maximum absolute value, $R^A_{i,j}$, of the waves within TW. The horizontal dotted lines represent the optimal threshold R^{th} found by Se/Sp analysis.

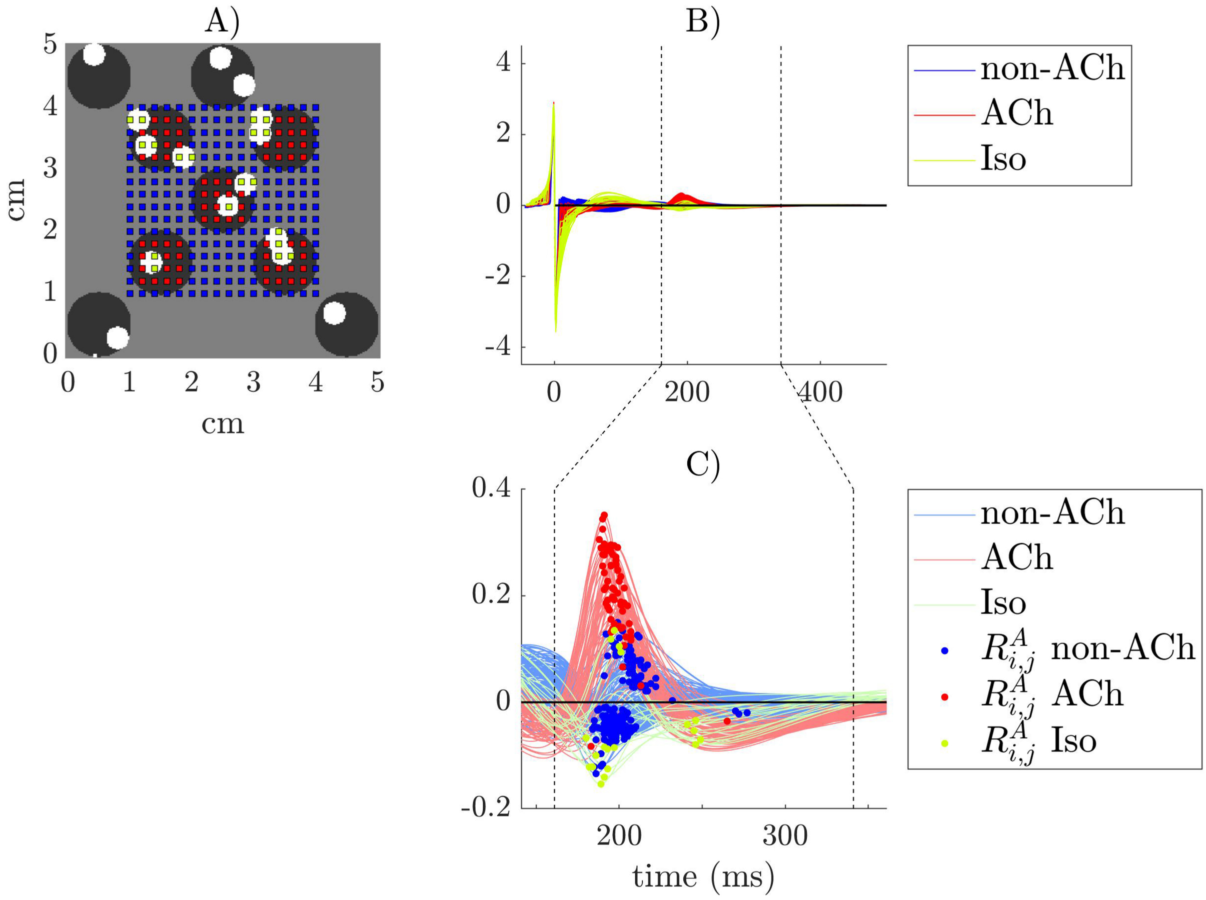

Figure S13: A) 2D model of a non-AF tissue with ACh release sites shown in black, β-adrenergically stimulated sites in white and EGM electrodes in red, blue and green. B) EGMs aligned with respect to the time correspondent to the maximum slope of the depolarization wave (marked as 0 in the x-axis). The vertical dashed lines delimit the time window TW for the $R_{i,j}(t)$ repolarization signals. C) Atrial repolarization waves, $R_{i,j}(t)$, with dots indicating the maximum absolute value, $R^A_{i,j}$, of the waves within TW.

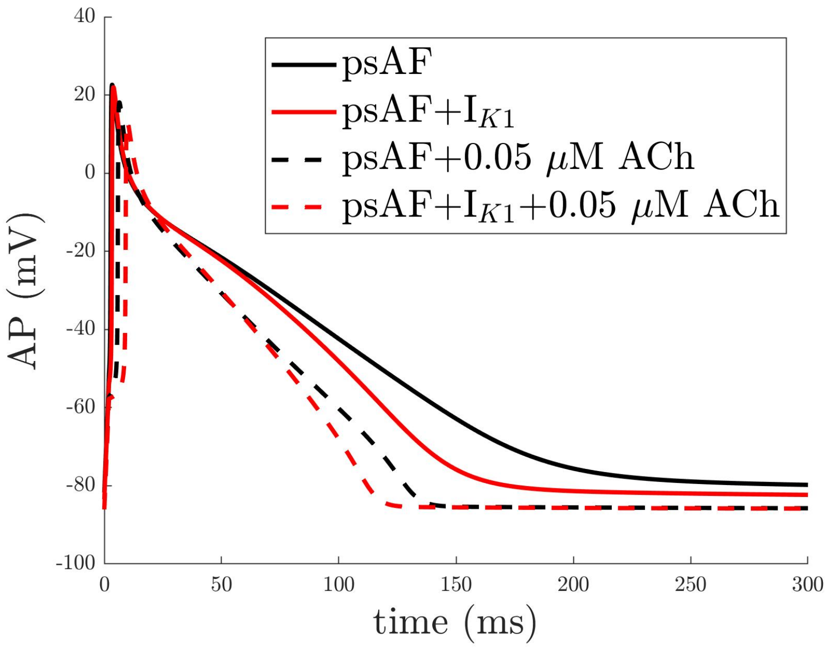

Figure S14: Action potentials obtained from single cell simulations pacing the cell at 1 Hz for 60 seconds. The continuous lines represent AP when ACh is not added, while the dashed lines represent the same cases with the addition of 0.05 μM ACh. The black lines represent the formulation of PsAF adopted in the manuscript, while the red lines represent the results after including 50% increase in I_{K1}.

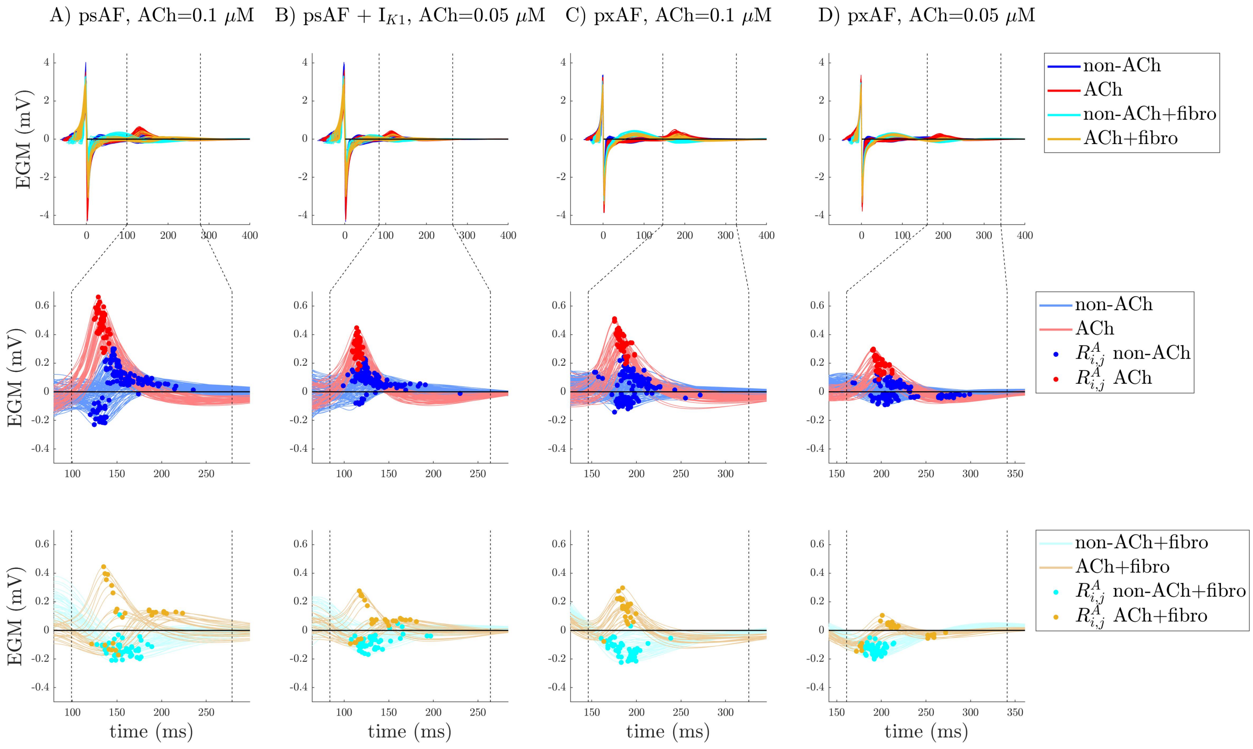

Figure S15: EGM analysis for the case with 0.5 cm ACh patches and 20% or 40% F^1_{nu}. Panel A) represents PsAF tissues without I_{K1} remodeling and ACh=0.1 μM. Panel B) represents PsAF tissues with I_{K1} remodeling and ACh=0.05 μM. Panel C) shows the PxAF case with ACh=0.1 μM. Panel D) corresponds to the PxAF case with ACh=0.05 μM. Top row: EGMs aligned with respect to the time correspondent to the maximum slope of the depolarization wave (marked as $t = 0$ in the x-axis). The vertical dashed lines delimit the time window TW for the $R_{i,j}(t)$ repolarization signals. Bottom row: atrial repolarization waves, $R_{i,j}(t)$, with dots indicating the maximum absolute value, $R^A_{i,j}$, of the waves within TW. The horizontal dotted lines represent the optimal threshold R^{th} found by Se/Sp analysis.
